# Is increased inflammation in schizophrenia associated with increased cardiovascular risk and the etiopathogenesis of this disorder?

**DOI:** 10.1016/j.bbih.2026.101370

**Published:** 2026-09-29

**Authors:** Muhammed Tuğrul Ergün, Memduha Aydın, Rukiye Tekdemir, Ömer Bayırlı, Kadri Murat Gürses, Muhammed Ulvi Yalçın, Halil Özalp, Hasibe Artaç, Ayça Ceylan, Sedat Abuşoğlu, Fikret Akyürek, Halil Güven, Yavuz Selvi, Mustafa Esad Tezcan

**Affiliations:** a Department of Psychiatry, Meram State Hospital, Konya, Türkiye; b Department of Psychiatry, Selçuk University Faculty of Medicine Hospital, Konya, Türkiye; c Department of Cardiology, Selçuk University Faculty of Medicine Hospital, Konya, Türkiye; d Department of Pediatric Allergy and Immunology, Selçuk University Faculty of Medicine Hospital, Konya, Türkiye; e Department of Biochemistry, Selçuk University Faculty of Medicine Hospital, Konya, Türkiye; f Department of Child and Adolescent Psychiatry, Selçuk University Faculty of Medicine Hospital, Konya, Türkiye

**Keywords:** Schizophrenia, Inflammation, NF-κB, Toll-like receptor, Cardiovascular disease, Cytokines

## Abstract

**Objective:**

This study aimed to examine the relationship between increased cardiovascular risk in schizophrenia and immune–inflammatory markers, including toll-like receptor (TLR)-2, TLR-4, and human leukocyte antigen (HLA)-DR expression on monocytes and their subsets, and to investigate the associations between the QRISK3 score and inflammatory markers.

**Methods:**

Serum cytokines (interleukin (IL)-1, IL-6, IL-10, tumor necrosis factor alpha (TNF-α), interferon gamma (IFN-γ), and nuclear factor kappa B (NF-κB)), monocyte subset distribution, and TLR-2/TLR-4/HLA-DR expression were assessed by flow cytometry in 77 individuals with schizophrenia and 50 healthy controls. Cardiovascular risk parameters, including left ventricular ejection fraction (LVEF), QRISK3, carotid intima-media thickness (CIMT), global longitudinal strain (GLS), and diastolic function, were evaluated using standard cardiological methods.

**Results:**

Compared with controls, the schizophrenia group showed higher IL-6 and IL-10 levels, lower NF-κB and IFN-γ levels, a higher proportion of classical and a lower proportion of non-classical monocytes. TLR-2 and TLR-4 expression differed across monocyte subsets, with higher mean fluorescence intensity (MFI) in classical and intermediate monocytes. They also had higher triglyceride (TG), QRISK3, and CIMT, but lower GLS and mitral E/A values. QRISK3 correlated positively with mean CIMT and negatively with IL-6 and TNF-α, while elevated IL-6 and reduced NF-κB were most strongly associated with schizophrenia in hierarchical regression analysis. In the regression analysis, however, QRISK3 showed no significant association with any inflammatory cytokine.

**Conclusion:**

Our findings suggest that individuals with schizophrenia may exhibit alterations in immune–inflammatory markers, monocyte subset distribution, TLR-2/TLR-4 and HLA-DR expression, as well as cardiovascular risk indicators. In addition, the identification of IL-6 and NF-κB as the biomarkers most strongly associated with schizophrenia, together with the positive association between IL-6 and CIMT, suggests their potential relevance.

## 1 Introduction

Schizophrenia is a chronic psychiatric disorder characterised by positive and negative symptoms accompanied by cognitive impairments; it affects emotion, thought, perception, and behaviour (McCutcheon et al., 2020). Schizophrenia is also associated with increased cardiovascular morbidity and mortality. These patients die 10–25 years earlier than the general population, and cardiovascular diseases (CVD) constitute the leading cause of death in schizophrenia (Olfson et al., 2015). The mechanisms underlying the increased susceptibility to CVD in schizophrenia are complex. The risk has been attributed not only to shared biological and genetic factors involved in the pathogenesis of the disorder but also to lifestyle factors affected by the illness and to treatment-related adverse effects (Badcock et al., 2019). These factors contribute to the development of several CVD risk conditions, most notably metabolic syndrome, and increase morbidity and mortality in the absence of adequate follow-up (Tam To et al., 2023).

Another key link between schizophrenia and CVD is immune dysfunction; immune dysregulation plays a significant role in the aetiology of schizophrenia. Although the mechanisms underlying immune dysregulation remain incompletely understood, studies have demonstrated alterations in both innate and adaptive cellular signalling pathways in schizophrenia (Cunha et al., 2016). In this context, studies have observed cytokine dysregulation in psychiatric disorders compared to healthy controls (Goldsmith et al., 2016). Atherosclerosis, which underlies the development of CVD, is currently recognised as an immuno-inflammatory disease. Therefore, the immune system plays a pivotal role in the development of both psychiatric disorders such as schizophrenia and CVD (Libby and Hansson, 2015). Moreover, immune dysregulation has been reported as a causal link between schizophrenia and CVD comorbidity (Morch et al., 2019). One study in this field reported that systemic inflammatory processes emerging in the early stages of psychosis, together with the resulting endothelial dysfunction, may create a proinflammatory microenvironment within the vessel wall, thereby forming the pathophysiological basis for the increased cardiovascular morbidity observed in these patients (Jameie et al., 2025). Among the primary cellular regulators of these inflammatory processes are nuclear factor kappa B (NF-κB) and toll-like receptors (TLRs). Activation of these pathways triggers the release of proinflammatory cytokines, thereby amplifying the inflammatory response at both the central nervous system and vascular levels. Therefore, NF-κB- and TLR-mediated signalling pathways are considered critical mediators in the biological link between schizophrenia and CVD (Mostafa et al., 2022).

NF-κB is one of the principal regulators of cellular inflammation. It regulates the expression of proinflammatory genes (Alharbi et al., 2021). NF-κB can be activated by both environmental and genetic factors and is capable of triggering the chronic inflammatory processes involved in the pathophysiology of schizophrenia (Venkatasubramanian and Debnath, 2013). TLRs are components of the innate immune system. They constitute a family of receptors capable of recognising microbial products and cellular damage signals (Lucas and Maes, 2013). The activation of TLRs enhances the expression of various proinflammatory cytokines by stimulating the NF-κB pathway, which plays a central role in intracellular signal transduction. Therefore, the TLR–NF-κB and associated cytokine pathway is considered a key regulatory mechanism in the initiation and maintenance of the immune system's inflammatory response. In this context, one study reported that monocyte TLR-2 levels in drug-naïve first-episode schizophrenia patients were comparable to those of healthy controls. However, a reduced response to monocyte stimulation has been demonstrated in schizophrenia (Juncal-Ruiz et al., 2020). A postmortem study demonstrated that the NF-κB system was suppressed across multiple brain regions in individuals with schizophrenia (Roussos et al., 2013). A recent meta-analysis reported that IL-1 and TNF-α levels were elevated during both the acute and chronic phases of schizophrenia compared to healthy controls (Halstead et al., 2023).

In conclusion, the TLR-2/4/NF-κB pathway plays a critical role in the pathogenesis of both schizophrenia and CVD, operating through cytokine-mediated and other immune mechanisms. The elevated CVD risk in individuals with schizophrenia is evident not only at the clinical level but also at biochemical and metabolic levels. In these patients, impaired lipid metabolism and reduced endothelial function have been reported alongside increased inflammatory activity (Joshi et al., 2013). The effects of immune-mediated inflammatory processes on lipid profiles and the vascular structure shed light on the biological mechanisms underlying the cardiovascular comorbidity observed in schizophrenia. In this context, the assessment of comprehensive cardiovascular risk scores such as QRISK3—which integrates multiple clinical variables to estimate 10-year CVD risk—alongside inflammatory markers may contribute to a better understanding of disease-specific immune–metabolic interactions.

These molecules were selected because TLR-2/TLR-4–mediated signalling, through NF-κB activation, constitutes a fundamental determinant of both peripheral and central immune responses. In addition to the chronic low-grade inflammation described in schizophrenia, cardiovascular processes such as atherosclerosis and endothelial dysfunction have been closely linked to these pathways (Goldsmith et al., 2016). Therefore, these biomarkers provide biologically meaningful targets for investigating the shared immune mechanisms underlying increased CVD risk in schizophrenia. Moreover, the TLR-2/TLR-4/NF-κB pathway is associated with processes such as microglial activation, oxidative stress, endothelial dysfunction, and synaptic remodeling. There is growing evidence that these mechanisms may play shared roles in both the pathophysiology of schizophrenia and cardiovascular comorbidities. This study aimed to evaluate these mechanisms collectively and investigate their potential interactions. The primary hypothesis of our study was that individuals with schizophrenia would exhibit differences in TLR-2/TLR-4 receptor expression, NF-κB levels, and associated cytokine profiles compared to healthy controls, and that these differences would be associated with systemic inflammation and CVD risk markers. Additionally, it was hypothesised that significant relationships would be identified between immune response indicators and clinical features, and that the biomarkers obtained could serve as potential predictors of schizophrenia.

To the best of our knowledge, no study has examined the potential relationships between increased CVD risk in schizophrenia and TLR-2/TLR-4/HLA-DR expression in monocyte subsets, NF-κB levels, associated cytokines, and QRISK3 scores. To address this gap, this study comprehensively investigates the potential impact of immune-mediated inflammation on CVD risk in schizophrenia and elucidates the clinical implications of this relationship.

## 2 Materials and methods

### 2.1 Participants

Individuals diagnosed with schizophrenia who presented to the Psychotic Disorders Outpatient Clinic of Selçuk University Faculty of Medicine (SUFM) Hospital were included in the study. Individuals with a total Positive and Negative Syndrome Scale (PANSS) score above 70 were excluded from the study. Individuals with schizophreniform disorder, acute psychotic disorder, schizoaffective disorder, bipolar disorder, autism spectrum disorder (ASD), intellectual disabilities, active infections, immunodeficiencies, or autoimmune or rheumatologic diseases were also excluded, along with those with a history of alcohol or substance dependence. For both the schizophrenia and healthy control groups, individuals with any infectious illness or use of anti-inflammatory medication within the previous month were excluded from the study. All diagnoses were confirmed by specialist psychiatrists using the Structured Clinical Interview for DSM-5 (SCID-5) based on DSM-5 criteria (Fig. 1).

**Fig. 1:**
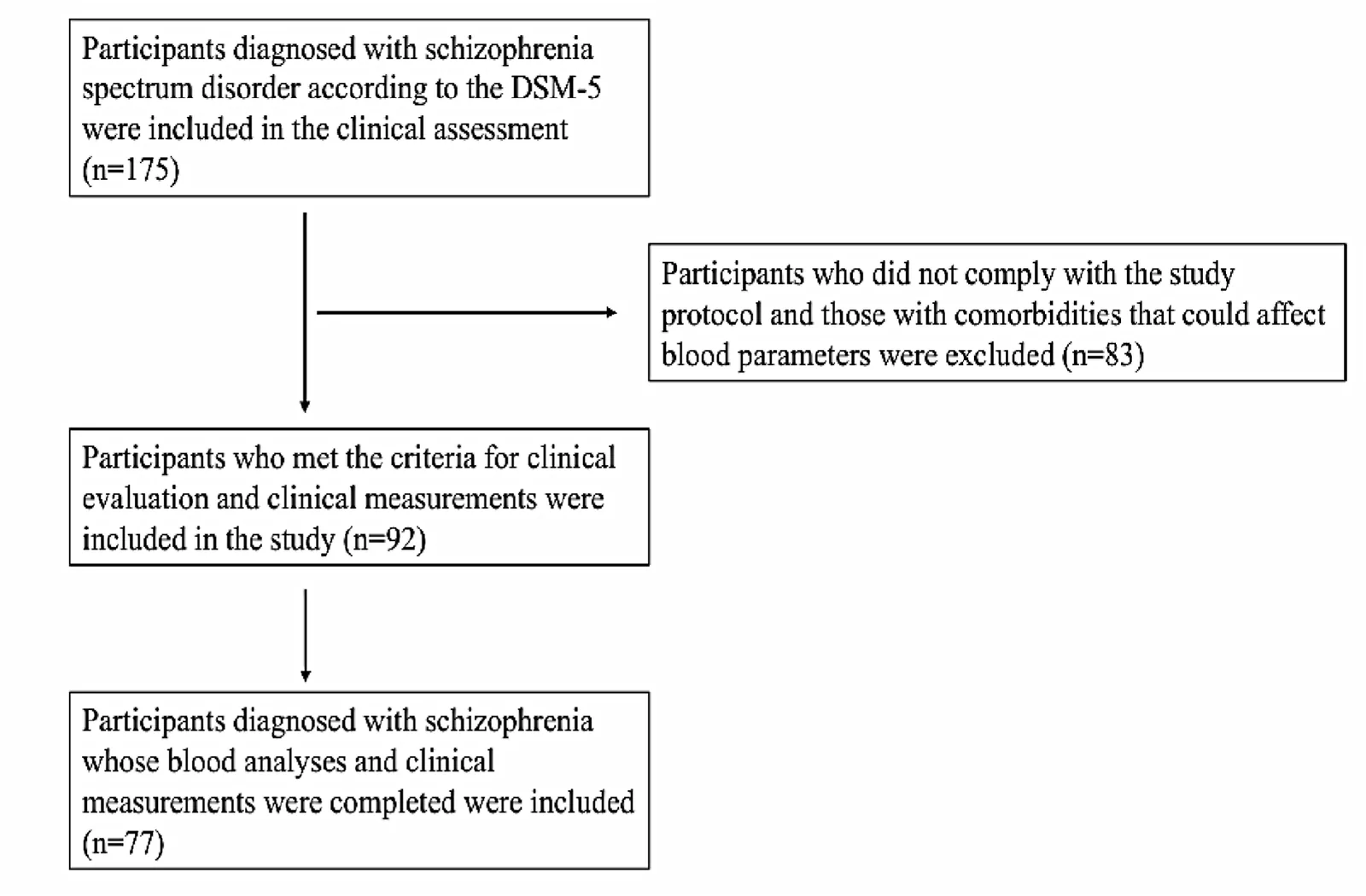
Flow chart of the inclusion process for participants diagnosed with schizophrenia.

The control group consisted of healthy individuals who presented to the clinic for consultation, had no psychiatric disorders according to the SCID-5 assessment, met the inclusion criteria, and were randomly selected after being matched with the patient group for age and sex. No neurological or systemic illnesses were present in the control group, and individuals receiving any regular medication were excluded from the study. A total of 77 individuals diagnosed with schizophrenia and 50 healthy controls aged 18–55 years were included in the study. Written and verbal informed consent was obtained from all participants. The study was conducted in accordance with the Declaration of Helsinki and approved by the Ethics Committee of SUFM on 16 July 2024 (decision no. 2024/368).

### 2.2 Clinical, physical, and cardiovascular assessments

The interviews were conducted by psychiatry specialists in accordance with the DSM-5 diagnostic criteria. The SCID-5 was administered to all participants to confirm psychiatric diagnoses or their absence. The SCID-5 is a clinician-administered structured diagnostic interview used to confirm existing psychiatric diagnoses in patients and the absence of psychiatric disorders in healthy individuals (First et al., 2015. In the validity and reliability study of the Turkish version of the SCID-5, the interrater agreement coefficient (κ) ranged from 0.49 to 1.00, indicating high concordance for most diagnoses (Elbir et al., 2019).

Participants diagnosed with schizophrenia were assessed using the PANSS and the Hamilton Depression Rating Scale (HDRS). The PANSS is a 30-item, clinician-rated scale used to assess symptom severity in schizophrenia (Kay et al., 1987). The total score reflects the overall level of symptom severity. Scores of 30–58 indicate minimal symptoms, 59–78 mild symptoms, 79–98 moderate symptoms, 99–118 marked symptoms, and ≥119 severe symptom levels (Kostakoğlu et al., 1999). The HDRS is a 17-item, semi-structured, clinician-administered scale used to assess the severity of depressive symptoms. A total score of 0–7 indicates a normal range, 8–13 indicates mild depression, 14–18 moderate, 19–22 severe, and ≥23 very severe levels of depression (Akdemir et al., 2001). Turkish validity and reliability studies showed that the overall internal consistency was approximately α ˜ 0.83 for the PANSS (Kostakoğlu et al., 1999) and α ˜ 0.75 for the HDRS (Akdemir et al., 2001).

In this study, the individuals in both groups also completed the Beck Anxiety Inventory (BAI), a 21-item self-report scale developed to measure the severity of anxiety symptoms (Beck et al., 1988). This scale was used during the diagnostic process and for monitoring treatment response. The reliability of the Turkish version is high (α ˜ 0.93) (Savaşır and Şahin, 1997). Following the assessment, the clinician completed the sociodemographic data forms for both groups to identify their clinical/demographic variables.

Physical measurement variables were recorded for the patients, including height (cm), weight (kg), blood pressure (mmHg), and waist circumference (cm). Cardiovascular parameters were assessed by an expert cardiologist in accordance with standard protocols. Endothelial function was measured using the brachial artery flow-mediated dilation (FMD) method with a 7–12 MHz linear probe. FMD was calculated as the percentage increase in arterial diameter following reactive hyperaemia relative to the baseline diameter. Left ventricular systolic function was assessed using speckle-tracking echocardiography, and global longitudinal strain (GLS) was determined from apical 4, 2, and long-axis views. Carotid intima–media thickness (CIMT) was calculated as the mean (mm) of the right and left measurements obtained from the proximal segment of the carotid bulb. Diastolic function was assessed using the mitral E/A and E/E′ ratios, while left ventricular ejection fraction (LVEF) was evaluated using the biplane Simpson method. Additionally, the 10-year cardiovascular risk of the participants was calculated using the QRISK3 algorithm. QRISK3 is a multivariable model developed to estimate individual CVD risk probability. This model incorporates variables such as severe mental illness and antipsychotic medication use, enabling a more comprehensive assessment of cardiovascular risk in schizophrenia (Hippisley-Cox et al., 2017).

### 2.3 Laboratory assessment

Blood samples were collected from all participants between 09:00 and 10:00 a.m. following at least 8 h of fasting. The samples were centrifuged at 4000 rpm for 10 min, and the sera were separated. They were stored at −80°C until ELISA analyses were performed. Levels of IL-1 (E0077H), IL-6 (E0090Hu), IL-10 (E0102Hu), TNF-α (E0082Hu), IFN-γ (E0105Hu), and NF-κB (E0690Hu) were analysed using ELISA kits (BTLAB, China) in accordance with the manufacturer's instructions. All samples were analysed in triplicate. The intra-assay coefficients of variation (CVs) were 5.1% for IL-1, 5.9% for IL-6, 5.8% for IL-10, 5.0% for TNF-α, 6.0% for IFN-γ, and 6.7% for NF-κB, whereas the inter-assay CV was <10% for all analytes. The analytical sensitivities were 0.54 pg/mL for IL-1, 1.03 ng/L for IL-6, 2.59 pg/mL for IL-10, 1.52 ng/L for TNF-α, 0.49 ng/mL for IFN-γ, and 0.01 ng/mL for NF-κB. The corresponding limits of quantification (LOQ) were 1 pg/mL, 2 ng/L, 5 pg/mL, 3 ng/L, 1 ng/mL, and 0.03 ng/mL, respectively. The NF-κB ELISA kit used in this study measures NF-κB protein but does not distinguish between individual NF-κB subunits (e.g., p65/RELA, p50/NFKB1, or p52/NFKB2); therefore, no subunit-specific analysis was performed. Colour changes generated in the ELISA kits were measured at 450 nm using a CLARIOstar Plus microplate reader. Sample concentrations were calculated using a calibration curve generated from standard concentrations and optical density values. Cytokine levels were determined using the ELISA method in the Medical Biochemistry Research Laboratory of SUFM.

The lipid parameters of the participants were measured in the Biochemistry Laboratory of SUFM using a Roche Cobas c702 analyser and Roche kits. Following calibration and internal quality controls, the results were calculated using a spectrophotometric method. Reference ranges were defined as 40–150 mg/dL for triglycerides (TG), 130–200 mg/dL for total cholesterol, <130 mg/dL for low density lipoprotein (LDL), and 40–60 mg/dL for high density lipoprotein (HDL).

Monocytes and their subsets, as well as TLR-2, TLR-4, and HLA-DR expression levels, were assessed by flow cytometry in blood samples obtained from the participants. One millilitre of peripheral blood collected into EDTA tubes (Becton Dickinson, CA, USA) was layered onto 500 μL of Histopaque-1077 (Sigma-Aldrich, USA) and centrifuged at 2000 rpm for 30 min. The isolated mononuclear cell layer was aspirated and washed with 1 mL of IsoFlow solution (Beckman Coulter, USA) at 1500 rpm for 5 min. Cells were stained with CD14 (PerCP), CD16 (PE/Cyanine-7), HLA-DR (FITC), CD282 (PE; TLR-2), and CD284 (APC; TLR-4) monoclonal antibodies (BioLegend, San Diego, CA, USA) and incubated in the dark for 20 min. After erythrocyte lysis with 500 μL of OptiLyse solution (Beckman Coulter, USA), flow cytometric acquisition was performed on a Beckman Coulter Navios flow cytometer, and data were analysed using Kaluza Analysis 2.1 software (Beckman Coulter, USA). At least 100,000 events were acquired for each sample.

Monocytes were initially identified on the basis of their forward scatter (FSC) and side scatter (SSC) characteristics. Doublets were excluded using a forward scatter peak (FS PEAK) versus forward scatter integral (FS INT) plot. Monocytes were subsequently classified according to CD14 and CD16 expression into classical (CD14++CD16^−^), intermediate (CD14++CD16^+^), and non-classical (CD14+CD16++) subsets (Fig. 2). The proportions of total monocytes and each monocyte subset were expressed as percentages (%), whereas HLA-DR, TLR-2, and TLR-4 expression levels were quantified as mean fluorescence intensity (MFI) in total monocytes and each monocyte subset. Appropriate fluorochrome-matched isotype controls were included to determine background fluorescence, verify staining specificity, and establish positive gates for HLA-DR, TLR-2, and TLR-4 expression.

**Fig. 2:**
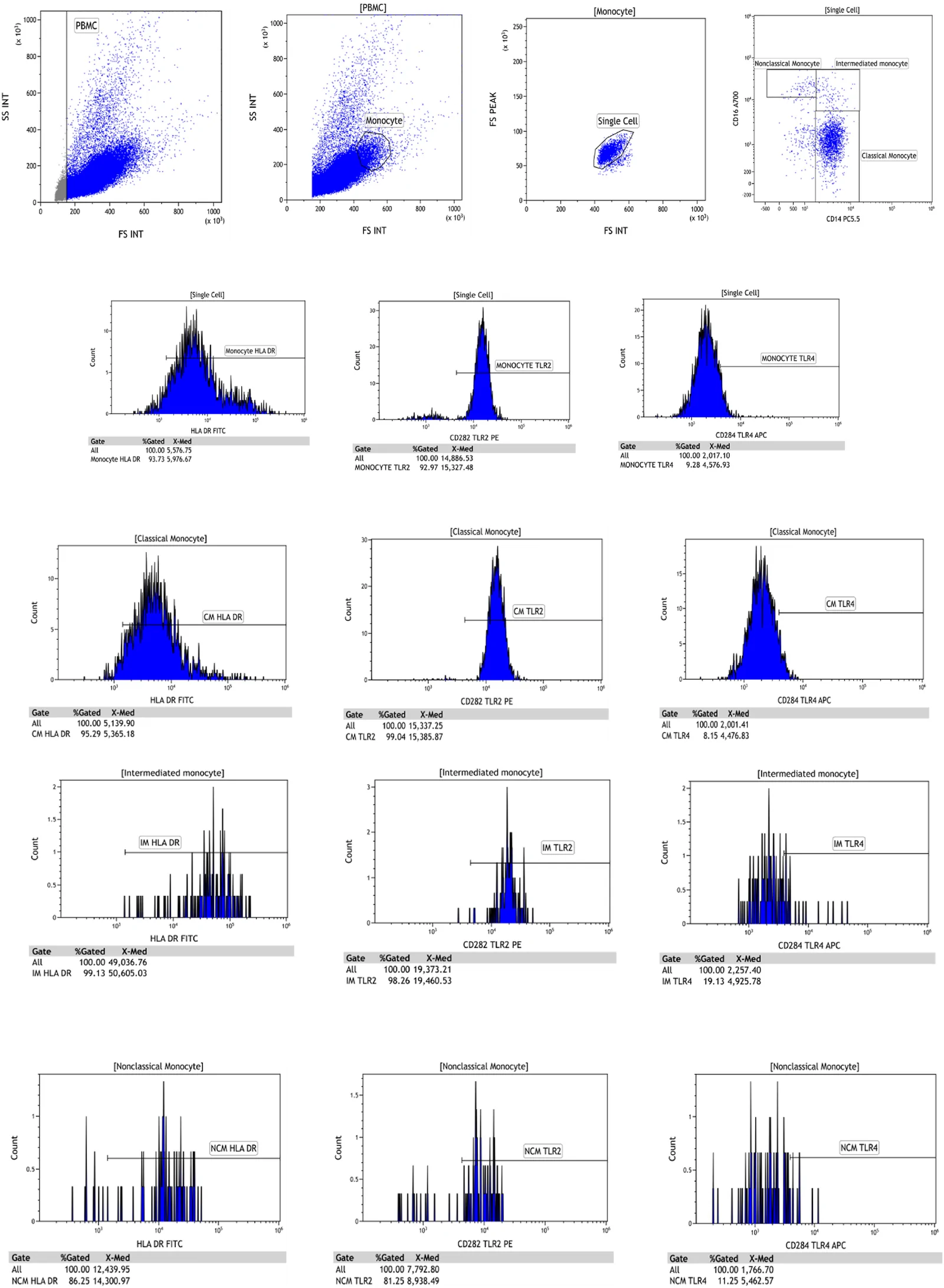
Gating strategy of monocyte subsets. Monocytes were gated in the myeloid population of the PBMCs on the FS–SS dot plot graph, then single cells were gated on the FS PEAK (height/amplitude)-FS INT (integral/area) plot to exclude doublet cells. Monocyte subsets were identified according to their CD14 and CD16 expressions. CL, classical monocytes; INT, intermediate monocytes; NC, non-classical monocytes; PBMC, peripheral blood mononuclear cell; SS, side scatter; FS, forward scatter; HLA-DR, human leukocyte antigen-DR locus; TLR, toll like receptor.

Fluorescence compensation was performed using single-stained compensation beads for each fluorochrome. Compensation matrices were generated automatically using Kaluza Analysis 2.1 software and were subsequently verified and manually adjusted by an experienced operator before data analysis. Daily instrument quality control was performed using Flow-Check Pro Fluorospheres (Beckman Coulter, USA) according to the manufacturer's recommendations to verify laser alignment, detector performance, and instrument stability. Identical instrument settings were maintained throughout the study to ensure the comparability of MFI measurements across all samples. Investigators performing flow cytometric acquisition and data analysis were blinded to participants' group allocation until completion of the statistical analyses.

### 2.4 Statistical analysis

Hierarchical logistic regression analyses were performed using Python (Python Software Foundation, https://www.python.org/), while Student's t-tests, Mann–Whitney U tests, and chi-square tests were conducted using SPSS 29.0 (SPSS Inc., Chicago, IL). The assumption of normality was evaluated based on skewness and kurtosis values falling within the range of −2 to +2 (George and Mallery, 2011). The results were presented with the model coefficient (β), standard error (SE), Wald Z statistic, p-value, and 95% confidence interval (CI), and a p-value <0.05 within the 95% CI was considered statistically significant.

Hierarchical logistic regression analysis was performed to evaluate the association of inflammatory markers and monocyte immunophenotyping variables with a diagnosis of schizophrenia. Because the cytokine distributions were skewed, regression models were fitted using log-transformed cytokine values. To improve the interpretability of the results and ensure numerical stability, continuous variables were standardized before being entered into the model. Accordingly, the odds ratios represent the change in the odds of schizophrenia associated with a one-standard-deviation increase in each variable. The regression analysis was conducted in two blocks. Model 1 included log-transformed peripheral inflammatory markers (IL-1, IL-6, IL-10, TNF-α, IFN-γ, and NF-κB). In Model 2, three monocyte subset-specific immunophenotyping variables were added to Model 1: classical monocyte TLR-2 MFI, intermediate monocyte HLA-DR MFI, and non-classical monocyte TLR-4 MFI. These variables were selected to represent different monocyte subsets and receptor axes while minimizing redundancy arising from the high correlations between total monocyte and subset-specific MFI measurements. Before model construction, multicollinearity was assessed using correlation matrices and variance inflation factors (VIFs). Highly overlapping variables were not entered into the same model simultaneously. In particular, total monocyte MFI measurements were found to be highly correlated with the corresponding classical monocyte MFI measurements, consistent with the overlap between the total monocyte gate and the subset-specific gates. Model fit and stability were evaluated using the −2 log likelihood (−2LL), Akaike information criterion (AIC), Bayesian information criterion (BIC), Nagelkerke R^2^, likelihood-ratio tests for nested models, receiver operating characteristic (ROC) curve analysis, the Hosmer–Lemeshow goodness-of-fit test, and VIFs.

Hierarchical multivariable linear regression analyses were performed to evaluate the independent association between inflammatory biomarkers and cardiovascular risk measures. Mean CIMT was used as the primary outcome variable, whereas the QRISK3 score was used as the secondary outcome variable. Distributional properties were examined before the analyses, and because IL-1, IL-6, IL-10, TNF-α, IFN-γ, and NF-κB levels were found to exhibit right-skewed distributions, these variables were log10-transformed. Mean CIMT was analysed in its original measurement unit. Separate hierarchical multiple linear regression models were constructed for mean CIMT and lg(QRISK3). In the first block, the diagnostic group and prespecified cardiovascular and echocardiographic covariates LVEF, absolute GLS, E/A ratio, E/e′ ratio, diastolic dysfunction, body mass index [BMI], waist circumference, systolic blood pressure, and diastolic blood pressure) were entered into the model simultaneously. In the second block, log10-transformed IL-1, IL-6, IL-10, TNF-α, IFN-γ, and NF-κB levels were entered into the model simultaneously. The additional contribution of the inflammatory biomarkers to the model was evaluated using the change in explained variance (ΔR^2^) and the corresponding F-change test. Because heteroscedasticity was detected in the residual analyses, HC3 heteroscedasticity-consistent standard errors and 95% confidence intervals were reported for coefficient-level inference in both outcome models. In addition, a robust joint Wald test was performed to evaluate whether the coefficients of the six biomarkers were jointly equal to zero. Multicollinearity was assessed using VIFs. The significance levels of the inflammatory biomarker coefficients were adjusted across the two primary regression models using the Benjamini–Hochberg false discovery rate (FDR) procedure. As part of the sensitivity analyses, the CIMT model was additionally adjusted for age and sex, whereas the QRISK3 analyses were repeated after excluding participants with documented cardiovascular disease. In addition, multivariable linear regression analyses were performed to evaluate the effect of chlorpromazine-equivalent doses on inflammatory cytokine levels.

To reduce the risk of type II errors, a multivariate analysis of covariance (MANCOVA) was applied for serum parameters, with age, BMI, sex, education level, marital status, socioeconomic status, and smoking status controlled as covariates. Before the analyses, parametric assumptions were met by applying logarithmic transformation to TLR-2 classical monocyte MFI, non-classical monocyte percentage, and TLR-4 non-classical monocyte MFI, and square root transformation to NF-κB levels. Since the MANCOVA results indicated significant between-group differences, a one-way analysis of covariance (ANCOVA) was conducted for each variable. Effect sizes were determined using Cohen's d for parametric and non-parametric comparisons and Cramér's V for categorical variables (≥0.8 large, 0.5–0.7 moderate, 0.2–0.4 small, <0.2 negligible) (Cohen, 2013). Relationships between serum parameters and clinical scales were evaluated using Pearson and Spearman correlations, and Bonferroni correction was applied to limit type I errors in multiple comparisons.

Considering the Cohen's d effect size, sample sizes, alpha level (α = 0.05) and intergroup sample ratio, post-hoc power was calculated for each morphometric measurement.

## 3 Results

A total of 127 participants were included in the study, comprising 77 individuals diagnosed with schizophrenia and 50 healthy controls. No significant differences were observed between the groups in terms of age (*t* = 0.474, *p* = 0.636), sex (χ^2^ = 0.189, *p* = 0.664), socioeconomic status (χ^2^ = 2.836, *p* = 0.242), or smoking status (χ^2^ = 5.377, *p* = 0.251). Significant differences were observed in education level (χ^2^ = 16.137, *p* < 0.001) and marital status (χ^2^ = 14.451, *p* = 0.001). BMI (*t* = 3.970, *p* < 0.001) and waist circumference (*t* = 5.246, *p* < 0.001) values were significantly higher in the schizophrenia group than the controls (Table 1).

**Table 1:** Clinical variables, demographic data, and blood parameter results for both groups.

	SCH (77)	HC (50)	*p*	*t/x*^*2*^*/z*	*d*
**Age**	36.88 ± 9.47	36.08 ± 9.09	0.636	0.474	0.396
					%7,6^g^
**Sex**^a^	Female (37)	Female (26)	0.664	0.189	0.039^a^
	Male (40)	Male (24)			
**Education Level**	Primary school (23)	Primary school (7)	**<0.001**	16.137	0.356^a^
	High school (25)	High school (6)			
	University (29)	University (37)			
**Socioeconomic Status**	<$500 (28)	<$500 (12)	0.242	2.836	0.149^a^
	$500–1000 (48)	$500–1000 (36)			
	>$1000 (1)	>$1000 (2)			
**Marital Status**	Single (44)	Single (15)	**0.001**	14.451	0.337^a^
	Married (19)	Married (29)			
	Divorced (14)	Divorced (6)			
**Cigarette Use (daily number of cigarettes)**	None (35)	None (33)	0.251	5.377	0.206
	Ex-smoker (5)	Ex-smoker (2)			
	<10 (4)	<10 (2)			
	11–20 (8)	11–20 (4)			
	>20 (25)	>20 (9)			
**BMI**	29.95 ± 5.41	26.35 ± 4.24	**<0.001**	3.970	**0.561**
**Waist Circumference**	100.83 ± 13.56	88.52 ± 11.86	**<0.001**	5.246	**0.784**
**LDL**^b^	117.83 ± 34.75	113.38 ± 31.01	0.775	−0.286	0.354
**HDL**^b^	46.67 ± 12.77	50.80 ± 13.05	0.059	−1.889	**3.613**
**TG**^b^	186.71 ± 131.30	116.50 ± 50.67	**0.001**	−3.363	**0.504**
**Cholesterol**	196.48 ± 34.65	185.70 ± 34.07	0.087	1.724	**0.571**
**HDRS**	7.90 ± 4.85	-	-	-	-
**PANSS-Positive**	9.69 ± 3.21	-	-	-	-
**PANSS-Negative**	13.32 ± 5.10	-	-	-	-
**PANSS-General**	24.44 ± 5.86	-	-	-	-
**PANSS-Total**	47.50 ± 11.77	-	-	-	-
**Total chlorpromazine-equivalent dose (mg/day)**	482,97 ± 315,78	-	-	-	-
**IL-1**^b^	67.75 ± 62.09	74.31 ± 74.66	0.643	−0.464	0.095
					%8,3^g^
**IL-6**^b^	224.40 ± 199.82	96.31 ± 108.93	**<0.001**^c^	−5.890	**4.590**
					**%98,4**^g^
**IL-10**^b^	420.34 ± 437.27	254.20 ± 291.46	**<0.001**^c^	−3.977	0.447
					%65,1^g^
**Tnf-α**^b^	190.20 ± 175.85	207.34 ± 173.20	0.073^c^	−1.794	0.098
					%8,3^g^
**INF-γ**^b^	75.30 ± 73.94	86.73 ± 63.59	0.039^c^	−2.068	0.165
					%14,5^g^
**NF-κB**^b^	2.61 ± 2.32	3.37 ± 1.98	**<0.001**	−3.494	**1.053**
					%47,3^g^
**Single Cell %**^b^	96.51 ± 2.51	95.93 ± 3.12	0.274	−1.093	**3.371**
					%21,3^g^
**Monocyte %**	3.90 ± 1.69	5.51 ± 3.30	**<0.001**^d^	3.610	0.164
					%94,8^g^
**Classical Monocyte %**	83.08 ± 6.54	80.24 ± 7.68	0.027^d^	−2.236	**1.051**
					%60,2^g^
**Intermediate Monocyte %**^b^	3.75 ± 2.98	3.66 ± 2.044	0.657^d^	−0.444	0.132
					%5,4^g^
**Non-classical Monocyte %**^b^	2.99 ± 1.58	3.65 ± 1.89	0.027^d^	−2.217	0.150
					%56,1^g^
**TLR-2 Monocyte %**^b^	90.95 ± 4.86	87.82 ± 7.20	0.005^e^	−2.789	**1.335**
					**%82,5**^g^
**TLR-2 Monocyte MFI**	15240.90 ± 2925.81	13637.66 ± 2376.98	**0.002**^e^	−3.241	0.060
					%89,5^g^
**TLR-2 Classical Monocyte %**^b^	98.33 ± 1.69	98.29 ± 1.50	0.820^e^	−0.227	**8.291**
					%5,2^g^
**TLR-2 Classical Monocyte MFI**^b^	15322.06 ± 2998.03	13720.88 ± 2396.93	**<0.001**^e^	−3.499	0.058
					%88,3^g^
**TLR-2 Intermediate Monocyte %**^b^	94.77 ± 6.48	94.29 ± 6.14	0.584^e^	−0.548	**1.350**
					%6,9^g^
**TLR-2 Intermediate Monocyte MFI**	20086.22 ± 3568.27	17068.62 ± 3372.07	**<0.001**^e^	−4.757	0.086
					%99,7^g^
**TLR-2 Non-classical Monocyte %**	64.41 ± 18.78	74.73 ± 13.77	**0.001**^e^	3.343	0.345
					%91,3^g^
**TLR-2 Non-classical Monocyte MFI**	11231.37 ± 2684.08	10030.08 ± 1896.03	0.007^e^	−2.749	0.051
					%77,9^g^
**TLR-4 Monocyte %**	9.56 ± 3.82	7.38 ± 3.40	**0.001**^e^	−3.285	0.262
					%90,3^g^
**TLR-4 Monocyte MFI**^b^	3675.83 ± 525.55	3864 ± 714.49	0.427^e^	−0.794	**0.585**
					%39,5^g^
**TLR-4 Classical Monocyte %**	8.39 ± 3.66	5.67 ± 2.76	**<0.001**^e^	−4.500	**0.422**
					**%99,4**^g^
**TLR-4 Classical Monocyte MFI**^b^	3622.88 ± 490.04	3759.38 ± 650.76	0.865^e^	−0.170	**0.563**
					%26,7^g^
**TLR-4 Intermediate Monocyte %**^b^	23.49 ± 12.16	19.56 ± 10.10	0.076^e^	−1.774	0.192
					%47,0^g^
**TLR-4 Intermediate Monocyte MFI**^b^	5327.02 ± 2274.39	5756.12 ± 2501.31	0.280^e^	−1.081	**1.987**
					%16,8^g^
**TLR-4 Non-classical Monocyte %**^b^	7.83 ± 8.41	6.04 ± 4.97	0.312^e^	−1.012	0.003
					%27,1^g^
**TLR-4 Non-classical Monocyte MFI**^b^	4503.02 ± 3243.30	4610.20 ± 1581.57	0.006^e^	−2.739	**1.746**
					%5,5^g^
**HLA-DR Monocyte %**^b^	91.19 ± 4.11	88.74 ± 5.03	**0.001**^e^	−3.238	**1.788**
					**%84,3**^g^
**HLA-DR Monocyte MFI**^b^	5423.06 ± 2959.34	4566 ± 1560.15	0.336^e^	−0.962	**2.290**
					%46,4^g^
**HLA-DR Classical Monocyte %**	95.02 ± 2.12	94.44 ± 2.73	0.185^e^	−1.331	**3.494**
					%26,2^g^
**HLA-DR Classical Monocyte HLA-DR MFI**^b^	4965.14 ± 2555.61	4134.26 ± 1399.88	0.161^e^	−1.401	**2.209**
					%55,0^g^
**HLA-DR Intermediate Monocyte %**^b^	97.55 ± 5.24	97.59 ± 5.14	0.806^e^	−0.246	**1.690**
					%5,0^g^
**HLA-DR Intermediate Monocyte MFI**	36556.49 ± 15523.01	30145.86 ± 12079.76	0.015^e^	−2.473	**0.460**
					%68,9^g^
**HLA-DR Non-classical Monocyte %**	73.43 ± 17.37	79.432 ± 13.79	0.042^e^	2.053	0.038
					%53,1^g^
**HLA-DR Non-classical Monocyte MFI**	14345.23 ± 6204.92	13244,54 ± 5522.06	0.310^e^	−1.019	0.018
					%17,3^g^
**LVEF**	60.81 ± 2.66	61.60 ± 2.49	0.100^f^	1.655	**2.352**
					%37,6^g^
**GLS**	17.62 ± 2.88	18.61 ± 2.50	0.049^f^	1.984	**0.583**
					%50,3^g^
**FMD**	8.26 ± 3.44	9.23 ± 4.09	0.149^f^	1.450	0.025
					%30,2^g^
**CIMT**	0.66 ± 0.10	0.57 ± 0.07	**<0.001**^f^	−5.312	**0.671**
					**%99,95**^g^
**Mitral Valve E/A Ratio**	1.06 ± 0.31	1.24 ± 0.26	**0.001**^f^	3.455	0.063
					%92,9^g^
**Mitral Valve E/E′ Ratio**	6.27 ± 1.46	6.76 ± 1.23	0.050^f^	1.977	0.036
					%50,1^g^
**Diastolic Dysfunction**	0.49 ± 0.50	0.18 ± 0.38	**<0.001**^f^	−3.740	0.109
					%96,3^g^
**QRISK3**^b^	4.43 ± 6.71	1.22 ± 1.71	**<0.001**^f^	−3.740	**0.904**
					**%90,67**^g^

**SCH:** schizophrenia, **HC:** healthy control, **BMI:** body mass index, **LDL:** low-density lipoprotein, **HDL:** high-density lipoprotein, **TG:** triglyceride, **HDRS**: Hamilton Depression Rating Scale, **PANSS:** Positive and Negative Syndrome Scale, **IL:** interleukin, **TNF-α:** tumour necrosis factor-alpha **INF-γ:** interferon gamma, **NF-κB:** nuclear factor kappa B, **TLR:** toll-like receptor, **HLA-DR:** human leukocyte antigen, **MFI:** mean fluorescence intensity, **LVEF:** left ventricular ejection fraction, **GLS:** global longitudinal strain, **FMD:** flow-mediated dilation, **QRISK3:** cardiovascular risk prediction score.

*d*: Cohen's d effect sizes.

a Cramer's V effect size.

b Mann-Whitney U.

c Bonferroni threshold: p < 0,0083.

d Bonferroni threshold: p < 0,0125.

e Bonferroni threshold: p < 0,0021.

f Bonferroni threshold: p < 0,00625.

g Post-hoc power test.

In the patient group, the mean age at first diagnosis was 23.69 ± 6.68 years, the mean disease duration was 13.19 ± 8.03 years, and the mean number of hospitalisations was 3.08 ± 2.63. Among participants with schizophrenia, the most commonly used antipsychotics were clozapine (n = 36, 46.8%) and paliperidone (n = 33, 42.9%). These were followed by aripiprazole (n = 18, 23.4%), olanzapine (n = 12, 15.6%), quetiapine (n = 11, 14.3%), risperidone (n = 3, 3.9%), haloperidol (n = 2, 2.6%), zuclopenthixol (n = 1, 1.3%), and amisulpride (n = 1, 1.3%). With regard to the number of antipsychotics used, 43 patients (55.8%) were receiving antipsychotic monotherapy, whereas 28 (36.4%) were receiving a combination of two antipsychotics and 6 (7.8%) were receiving a combination of three antipsychotics. No patients were receiving four or more antipsychotics. When evaluated according to antipsychotic class, 74 patients (96.1%) were receiving second-generation antipsychotic treatment only, whereas 3 (3.9%) were receiving a combination of first- and second-generation antipsychotics. No patients were receiving first-generation antipsychotic monotherapy.

Examination of the biochemical parameters revealed no significant differences between the groups in terms of LDL (*t* = −0.286, *p* = 0.775), HDL (*t* = −1.889, *p* = 0.059), or total cholesterol (*t* = 1.724, *p* = 0.087), whereas TG levels were significantly higher in the schizophrenia group compared with the controls (*t* = −3.363, *p* = 0.001) (Table 1).

When inflammatory markers were compared between the two groups, no significant differences were found for IL-1 (*z* = −0.464, *p* = 0.643) or TNF-α (*z* = −1.794, *p* = 0.073), whereas IL-6 (*z* = −5.890, *p* < 0.001) and IL-10 (*z* = −3.977, *p* < 0.001) levels were markedly higher in the schizophrenia group compared with the controls. IFN-γ (*z* = −2.068, *p* = 0.039) and NF-κB (*z* = −3.494, p < 0.001) levels were significantly lower in the schizophrenia group compared with the controls (Table 1). Box plots of inflammatory variables for both groups are presented in Fig. 3.

**Fig. 3:**
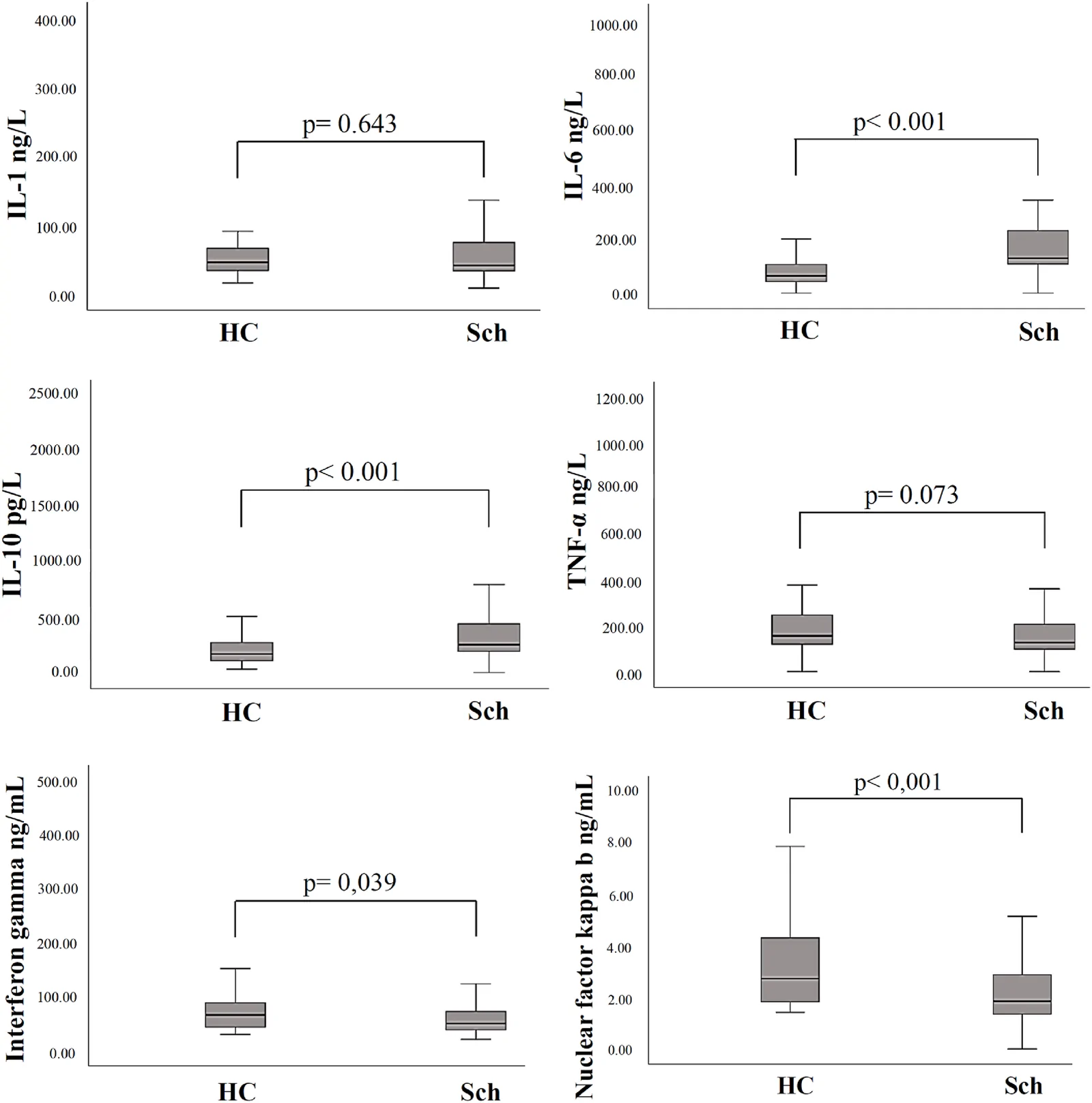
Box plots of blood parameters for both groups. **SCH:** schizophrenia, **HC:** healthy controls, **IL:** interleukin, **TNF-α:** tumour necrosis factor-alpha, **INF-γ:** interferon gamma.

In the analyses of monocyte subsets, the monocyte percentage was found to be significantly lower in the schizophrenia group (*z* = 3.610, *p* < 0.001). The percentage of classical monocytes was higher in the schizophrenia group (*z* = −2.236, *p* = 0.027), whereas the percentage of non-classical monocytes was significantly lower (*z* = −2.217, *p* = 0.027). No significant difference was observed in the percentage of intermediate monocytes (*z* = −0.444, *p* = 0.657).

With regard to TLR-2 expression, the TLR-2 monocyte percentage (*z* = −2.789, *p* = 0.005), TLR-2 monocyte MFI (*z* = −3.241, *p* = 0.002), TTLR-2 classical monocyte MFI (*z* = −3.499, *p* < 0.001), TLR-2 intermediate monocyte MFI (*z* = −4.757, *p* < 0.001), and TLR-2 non-classical monocyte MFI (*z* = −2.749, *p* = 0.007) were all significantly higher in the schizophrenia group.

In contrast, the percentage of TLR-2 non-classical monocytes was found to be significantly lower in the schizophrenia group (*z* = 3.343, *p* = 0.001). No significant differences were observed in the percentages of TLR-2 classical and intermediate monocytes (*p* > 0.05). For TLR-4 parameters, the schizophrenia group showed significantly higher TLR-4 monocyte percentage (*z* = −3.285, *p* = 0.001), TLR-4 classical monocyte percentage (*z* = −4.500, *p* < 0.001), and TLR-4 non-classical monocyte MFI levels (*z* = −2.739, *p* = 0.006). No significant differences were observed between the two groups for the remaining TLR-4 parameters (*p* > 0.05).

For HLA-DR expression parameters, the total HLA-DR monocyte percentage (*z* = −3.238, *p* = 0.001) and HLA-DR intermediate monocyte MFI (*z* = −2.473, *p* = 0.015) were found to be elevated in the schizophrenia group. In contrast, the percentage of HLA-DR non-classical monocytes was found to be significantly lower in the schizophrenia group (*z* = 2.053, *p* = 0.042). No significant differences were observed in the other HLA-DR parameters (monocyte MFI, and classical and intermediate percentages) (*p* > 0.05) (Table 1).

With regard to cardiac and vascular indicators, no significant differences were observed between the two groups in terms of LVEF (*t* = 1.655, *p* = 0.100) or FMD (*t* = 1.450, *p* = 0.149). GLS values were significantly lower in the schizophrenia group compared with the controls (*t* = 1.984, *p* = 0.049), while CIMT was markedly increased (*t* = −5.312, *p* < 0.001). The mitral E/A ratio (*t* = 3.455, *p* = 0.001) and the E/E ratio (*t* = 1.977, *p* = 0.050) in the schizophrenia group differed from the controls in a direction indicative of diastolic dysfunction. The presence of diastolic dysfunction (*z* = −3.740, *p* < 0.001) and the QRISK3 cardiovascular risk score (*z* = −3.740, *p* < 0.001) were also found to be significantly higher in the schizophrenia group (Table 1).

The MANCOVA demonstrated a significant difference between the groups (Pillai's trace *V=*0.999, *F*_(14.103)_ = 9486.081, *p* < 0.001, ηp^2^ = 0.999). Subsequently, an ANCOVA was conducted using the same covariates, and the differences between the groups are presented in Table 2. Following the ANCOVA analyses, the results remained unchanged, with monocyte percentage, TLR-2 non-classical monocyte percentage, and HLA-DR non-classical monocyte percentage being significantly lower in the schizophrenia group [*F*_(1.116)_ = 8.516, *p* = 0.004, ηp^2^ = 0.126; *F*_(1.116)_ = 10.210, *p* = 0.002, ηp^2^ = 0.146; *F*_(1.116)_ = 6.452, *p* = 0.012, ηp^2^ = 0.109, respectively]. Conversely, Log TLR-2 classical monocyte MFI, TLR-2 monocyte MFI, TLR-4 monocyte percentage, TLR-4 classical monocyte percentage, HLA-DR intermediate monocyte MFI, and TLR-2 intermediate monocyte MFI levels were significantly higher [*F*_(1.116)_ = 8.516, *p* = 0.004, ηp^2^ = 0.126; *F*_(1.116)_ = 5.226, *p* = 0.024, ηp^2^ = 0.111; *F*_(1.116)_ = 4.882, *p* = 0.029, ηp^2^ = 0.205; *F*_(1.116)_ = 12.353, *p* = 0.001, ηp^2^ = 0.236; *F*_(1.116)_ = 6.990, *p* = 0.009, ηp^2^ = 0.170; *F*_(1.116)_ = 18.547, *p* < 0.001, ηp^2^ = 0.193, respectively]. The blood parameters evaluated by controlling for age, BMI, sex, education level, marital status, socioeconomic status, and smoking are presented in Table 2.

**Table 2:** Comparison of blood parameters between groups according to ANCOVA.

Total Samples ANCOVA^a^	SCH (77)		HC (50)		*F* (1.116)	*p*	η_p_^2^	OP
	Mean	SD	Mean	SD				
**Monocyte %**	3.90	1.69	5.51	3.30	8.516	**0.004**	0.126	0.825
**Classical Monocyte %**	83.08	6.54	80.24	7.68	3.932	0.050	0.078	0.503
**Log Non-classical Monocyte %**	0.41	0.23	0.50	0.24	3.400	0.068	0.097	0.448
**TLR-2 Monocyte MFI**	15240.90	2925.81	13637.66	2376.98	5.226	**0.024**	0.111	0.621
**Log TLR-2 Classical Monocyte MFI**	4.1770	0.08	4.13	0.071	4.478	**0.036**	0.103	0.555
**TLR-2 Intermediate Monocyte MFI**	20086.22	3568.27	17068.62	3372.07	18.547	**0.000**	0.193	0.990
**TLR-2 Non-classical Monocyte %**	64.41	18.78	74.73	13.77	10.210	**0.002**	0.146	0.887
**TLR-2 Non-classical Monocyte MFI**	11231.37	2684.08	10030.08	1896.03	3.918	0.050	0.145	0.501
**TLR-4 Monocyte %**	9.56	3.82	7.38	3.40	4.882	**0.029**	0.205	0.591
**TLR-4 Classical Monocyte %**	8.39	3.66	5.67	2.76	12.353	**0.001**	0.236	0.936
**Log TLR-4 Non-classical Monocyte MFI**	3.60	0.17	3.64	0.14	0.645	0.423	0.072	0.125
**HLA-DR Intermediate Monocyte MFI**	36556.49	15523.01	30145.86	12079.76	6.990	**0.009**	0.170	0.746
**HLA-DR Non-classical Monocyte %**	73.43	17.37	79.43	13.79	6.452	**0.012**	0.109	0.712
**Sqrt NF-κB**	1.47	0.66	1.77	0.48	3.844	0.052	0.136	0.494

**SCH:** schizophrenia **HC:** healthy control, **SQRT:** square root., **Log:** logarithm, **OP:** observed power, **NF-κB:** nuclear factor kappa B, **TLR:** toll-like Receptor, **MFI:** mean fluorescence intensity, **CD:** cluster of differentiation**, HLA:** human leukocyte antigen, **SD:** standard deviation, **ANCOVA:** analysis of covariance.

a Covariates: age, BMI, sex, education level, marital status, socioeconomic status, smoking status.

In the hierarchical regression model performed to examine the association between peripheral blood markers and schizophrenia, Model 1 provided a significantly better fit than the null model: χ^2^(6) = 115.27, p < 0.001. The additional contribution of Model 2 beyond Model 1 was not significant: Δχ^2^(3) = 2.48, p = 0.479. According to these results, the cytokine model was able to discriminate the diagnostic group effectively; however, when the selected monocyte MFI variables were added to the model, they did not provide a statistically significant additional explanatory contribution (Table 3 and Fig. 4).

**Table 3:** Hierarchical regression analysis findings for inflammatory markers.

Model	Variables	−2LL	AIC	BIC	Nagelkerke R^2^	AUC	Hosmer–Lemeshow p	Accuracy	AUC %95 CI
Null model	Intercept	170,28	172,28	175,12	—	—	—	—	-
Model 1	IL-1, IL-6, IL-10, TNF-α, IFN-γ, NF-κB	55,01	69,01	88,92	0,808	0,981	0,282	%96,1	0,950–0,999
Model 2	Model 1 + classical TLR-2 MFI + intermediate HLA-DR MFI + non- classical TLR-4 MFI	52,53	72,53	100,97	0,818	0,982	0,094	%96,1	0,952–0,999

**−2LL:** −2 log likelihood; **AIC:** Akaike information criterion; **BIC:** Bayesian information criterion; **AUC:** area under the curve; **CI:** confidence interval; **MFI:** median fluorescence intensity; **HLA-DR:** human leukocyte antigen-DR; **TLR:** toll-like receptor; **NF-κB:** nuclear factor kappa B; **IL:** interleukin; **TNF-α:** tumor necrosis factor alpha; **IFN-γ:** interferon gamma.

**Fig. 4:**
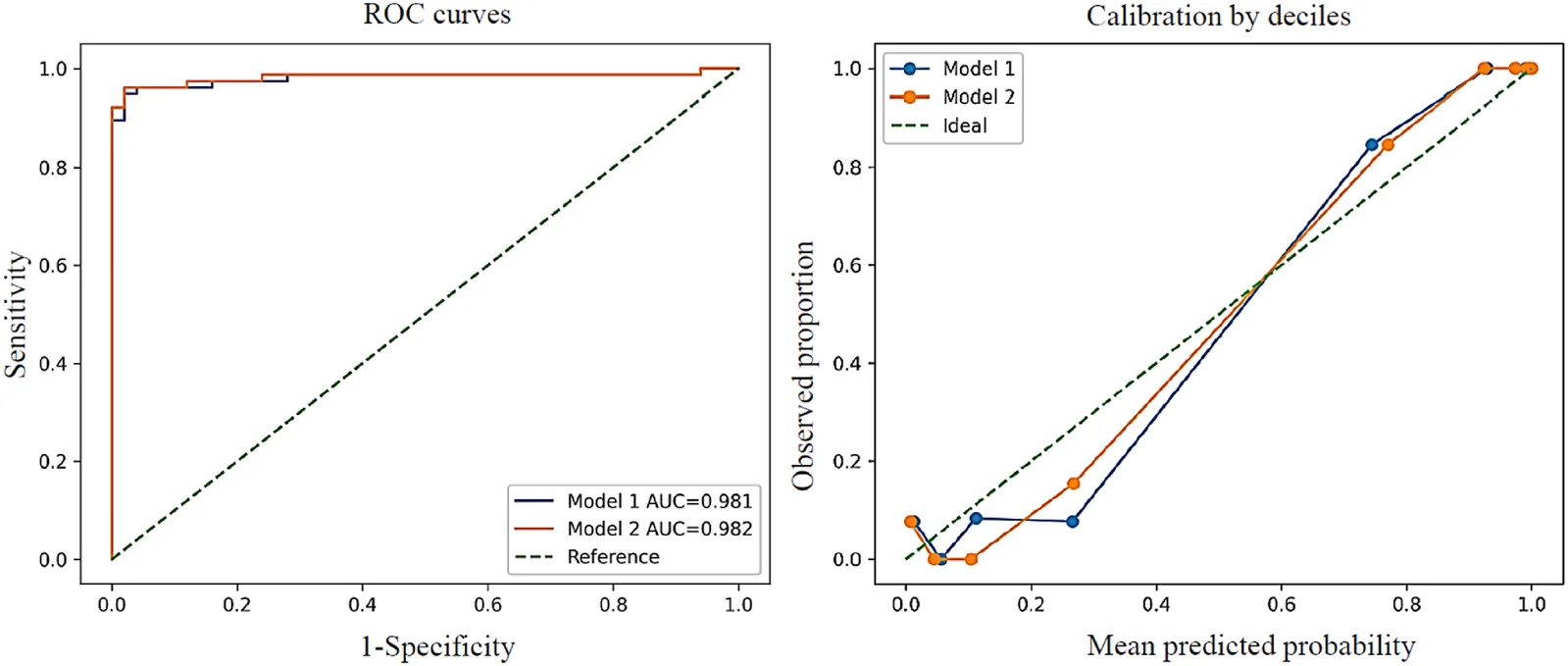
ROC and calibration results of the hierarchical regression model.

As described above, the hierarchical logistic regression analysis including both groups was conducted in two models. For Model 1, which included log-transformed IL-1, IL-6, IL-10, TNF-α, IFN-γ, and NF-κB levels, the −2 log-likelihood value was 55.01, the Nagelkerke R^2^ value was 0.808, and the AUC was 0.981. In this model, higher IL-6 levels, higher IL-10 levels, and lower NF-κB levels were independently associated with a diagnosis of schizophrenia.

In Model 2, classical monocyte TLR-2 MFI, intermediate monocyte HLA-DR MFI, and non-classical monocyte TLR-4 MFI were added to the cytokine model. For Model 2, the −2 log-likelihood value was 52.53, the Nagelkerke R^2^ value was 0.818, and the area under the curve (AUC) was 0.982. However, the additional contribution of Model 2 beyond Model 1 was not statistically significant (Δχ^2^(3) = 2.48, p = 0.479). In addition, the inclusion of the monocyte MFI variables increased the AIC and BIC values, suggesting that these variables did not provide a strong and statistically significant additional explanatory contribution beyond the cytokine model. In Model 2, IL-6 and NF-κB remained the strongest biomarkers independently associated with a diagnosis of schizophrenia, whereas none of the selected monocyte MFI variables was independently associated with the diagnosis. In the multicollinearity analysis, no collinearity of a magnitude likely to pose a problem was detected among the selected monocyte MFI variables; the VIF values for these variables ranged from 1.04 to 1.24. The highest VIF value observed in the final model was 5.68. The Hosmer–Lemeshow goodness-of-fit test showed no significant lack of fit for either Model 1 or Model 2. In the hierarchical logistic regression analysis, the cytokine-based model effectively discriminated schizophrenia diagnosis, and this effect was primarily driven by IL-6 and NF-κB. The addition of the selected monocyte MFI variables did not significantly improve model fit, and none of these variables was independently associated with the diagnosis after accounting for the cytokine markers (Table 4).

**Table 4:** Regression model results for inflammatory markers.

Variables	Model 1 OR	%95 CI	p	Model 2 OR	%95 CI	Model 2 VIF	p
lg IL-1	0,54	0,09–3,36	0,511	0,44	0,06–3,18	5,68	0,418
lg IL-6	90,02	12,40–653,66	**<0,001**	98,25	12,33–782,88	2,01	**<0,001**
lg IL-10	3,80	1,08–13,36	0,037	4,03	1,16–13,97	2,34	**0,028**
lg TNF-α	0,97	0,45–2,08	0,930	1,03	0,45–2,34	1,79	0,943
lg IFN-γ	0,31	0,03–2,94	0,308	0,26	0,02–2,84	5,46	0,271
lg NF-κB	0,006	0,0002–0,228	**0,006**	0,006	0,0002–0,212	1,81	**0,005**
Classical monocytes TLR-2 MFI	—	—	—	0,96	0,41–2,25	1,24	0,931
Intermediate monocytes HLA-DR MFI	—	—	—	1,81	0,83–3,93	1,20	0,135
Non- classical monocytes TLR-4 MFI	—	—	—	0,88	0,44–1,76	1,04	0,723

**OR:** odds ratio; **CI:** confidence interval; **VIF:** variance inflation factor; **IL:** interleukin; **TNF-α:** tumor necrosis factor alpha; **IFN-γ:** interferon gamma; **NF-κB:** nuclear factor kappa B; **HLA-DR:** human leukocyte antigen-DR; **TLR-2:** toll-like receptor 2; **TLR-4:** toll-like receptor 4; **MFI:** median fluorescence intensity. **Lg:** indicates log-transformed values.

Correlation analyses were performed with the biomarkers included in the hierarchical regression model. To avoid potential type I errors arising from multiple correlation analyses, Bonferroni correction was applied. Accordingly, a negative correlation was observed between TNF-α and mean CIMT levels (*r* = −0.416, *p* < 0.001), but no significant correlations were detected among the other variables (Table 5, Table 6).

**Table 5:** Correlation coefficients between blood parameters and clinical severity.

		HDRS	PANSS-P	PANSS-N	PANSS-G	PANSS-T	BAI
**IL-1**	***p***	0.322	0.925	0.528	0.810	0.628	0.711
	***r***	−0.114	−0.011	−0.073	0.028	−0.056	0.043
**IL-6**	***p***	0.166	0.740	0.472	0.852	0.432	0.493
	***r***	−0.159	−0.038	−0.083	−0.022	−0.091	−0.079
**IL-10**	***p***	0.433	0.751	0.326	0.834	0.735	0.673
	***r***	−0.091	0.037	−0.114	0.024	−0.039	0.049
**Tnf*-*α**	***p***	0.616	0.564	0.232	0.394	0.787	0.488
	***r***	−0.058	0.067	−0.138	0.098	−0.031	0.080
**INF-γ**	***p***	0.100	0.371	0.485	0.643	0.233	0.701
	***r***	−0.189	−0.103	−0.081	−0.054	−0.138	0.045
**NF-κB**	***p***	0.043	0.095	0.103	0.157	0.023	0.740
	***r***	−0.232	−0.192	−0.187	−0.163	−0.259	−0.038
**TLR-2 Classical Monocyte MFI**	***p***	0.019	0.021	0.405	0.086	0.060	0.797
	***r***	−0.266	−0.262	−0.096	−0.197	−0.216	−0.030
**TLR-4 Non-classical Monocyte MFI**	***p***	0.664	0.907	0.623	0.409	0.321	0.766
	***r***	−0.050	−0.014	−0.057	−0.096	−0.115	−0.034
**HLA-DR Intermediate Monocyte MFI**	***p***	0.071	0.002	0.553	0.070	0.042	0.459
	***r***	−0.207	−0.352	−0.069	−0.208	−0.233	−0.086

**IL:** interleukin, **TNF-α:** tumour necrosis factor-alpha**, INF-γ:** interferon gamma**, NF-κB:** nuclear factor kappa B, **TLR:** toll-like receptor**, HLA:** human leukocyte antigen, **MFI:** mean fluorescence intensity, **PANSS:** positive and negative syndrome scale. In this table, results with a Bonferroni-corrected p < 0.00043 were considered statistically significant.

**Table 6:** Correlation coefficients between blood parameters and cardiological data.

		LVEF	FMD	CIMT	GLS	E/A Ratio	E/E Ratio	Diastolic Dysfunction
**IL-1**	***p***	0.915	0.736	0.112	0.960	0.740	0.458	0.511
	***r***	−0.012	0.039	−0.183	0.006	0.038	−0.086	0.076
**IL-6**	***p***	0.969	0.246	0.008	0.788	0.467	0.169	0.479
	***r***	−0.005	−0.134	−0.302	0.031	0.084	−0.158	−0.082
**IL-10**	***p***	0.521	0.414	0.035	0.853	0.182	0,164	0.515
	***r***	0.074	−0.095	−0.240	0.022	0.154	−0.160	−0.075
**TNF-α**	***p***	0.905	0.251	**0.000**	0.274	0.375	0.275	0.525
	***r***	−0.014	−0.132	**−0.416**	−0.126	0.103	−0.126	−0.074
**INF-γ**	***p***	0.917	0.867	0.189	0.967	0.474	0.515	0.904
	***r***	0.012	−0.019	−0.151	0.005	0.083	−0.075	0.014
**NF-κB**	***p***	0.774	0.251	0.038	0.848	0.863	0.396	0.511
	***r***	0.033	−0.132	−0.237	−0.022	0.020	−0.098	0.076
**TLR-2 Classical Monocyte MFI**	***p***	0.876	0.906	0.872	0.772	0.821	0.953	0.464
	***r***	0.018	−0.014	−0.019	−0.034	0.026	0.007	−0.085
**HLA-DR Intermediate Monocyte MFI**	***p***	0.529	0.511	0.890	0.850	0.213	0.152	0.436
	***r***	−0.073	0.076	−0.016	0.022	0.144	−0.165	−0.090
**TLR-4 Non-classical Monocyte MFI**	***p***	0.934	0.614	0.117	0.165	0.281	0.280	0.458
	***r***	−0.010	0.058	−0.180	0.160	0.124	0.125	−0.086

**IL:** interleukin, **TNF-α:** tumour necrosis factor-alpha**, INF-γ:** interferon gamma**, NF-κB:** nuclear factor kappa B, **TLR:** toll-like receptor**, HLA:** human leukocyte antigen, **MFI:** mean fluorescence intensity, **CIMT:** carotis intima–media thickness, **LVEF:** left ventricular ejection fraction, **GLS:** global longitudinal strain, **FMD:** flow-mediated dilation. In this table, results with a Bonferroni-corrected p < 0.00043 were considered statistically significant.

In our study, QRISK3 was calculated in individuals with schizophrenia, and correlations between QRISK3, other cardiological measures, and the blood parameters included in the hierarchical regression analysis were also evaluated. After Bonferroni correction, QRISK3 was found to be positively correlated with mean CIMT (r = 0.518, p < 0.001) and negatively correlated with IL-6 and TNF-α (r = −0.424, p < 0.001; r = −0.403, p < 0.001, respectively) (Table 7).

**Table 7:** Correlation coefficients between QRISK and cardiological variables and inflammatory markers.

		**LVEF**	**FMD**	**CIMT**	**GLS**	**E/A Ratio**	**E/E Ratio**	**Diastolic Dysfunction**		

**QRISK3**	***p***	0.109 −0.184	0.749	**0.000**	0.796 −0.030	0.004 −0.321	0.123 −0.177	0.028		
	***r***		−0.037	**−0.518**				−0.251		
		**IL-1**	**IL-6**	**IL-10**	**Tnf-α**	**INF-γ**	**NF-κB**	**CM**	**IM**	**NM**

**QRISK3**	***p***	0.062	**0.000**	0.009	**0.000**	0.066	0.018	0.781	0.263	0.587
	***r***	−0.214	**−0.424**	−0.295	**−0.403**	−0.211	−0.269	−0.032	−0.129	−0.063

**LVEF:** left ventricular ejection fraction, **GLS:** global longitudinal strain, **FMD:** flow-mediated dilation, **QRISK3:** cardiovascular risk prediction score, **CM**: classical monocyte, **IM:** intermediate monocyte, **CIMT**: carotis intima–media thickness, **NM:** non-classical monocyte. In this table, results with a Bonferroni-corrected p-value <0.0031 were considered statistically significant.

Following the correlation analysis, multivariable regression analyses were performed to examine the relationship between inflammatory markers and cardiovascular risk measures, with QRISK3 or CIMT used as the dependent variable. The regression modeling was performed in two stages. First, only inflammatory cytokines from participants with schizophrenia were included in the model, whereas in the second model, cytokines from participants in both groups were included. The model including all biomarkers was statistically significant for CIMT but not for QRISK3. This finding suggests that a substantial proportion of the variation in CIMT and QRISK3 may be associated with differences between the diagnostic groups rather than with cytokines themselves (Table 8).

**Table 8:** Regression models according to cardiovascular risk measures.

Dependent variable	Model	R^2^	Adjusted R^2^	Model F	Model p	Max. VIF
CIMT	Stage 1: Inclusion of inflammatory biomarkers in the schizophrenia group	0,106	0,062	2379	**0,033**	5,47
CIMT	Stage 2: Inclusion of inflammatory biomarkers from both groups	0,204	0,157	4361	**<0,001**	6,18
QRISK3	Stage 1: Inclusion of inflammatory biomarkers in the schizophrenia group	0,080	0,034	1730	0,120	5,47
QRISK3	Stage 2: Inclusion of inflammatory biomarkers from both groups	0,173	0,124	3543	**0,002**	6,18

**CIMT:** carotid intima–media thickness; **VIF:** variance inflation factor; **QRISK3:** cardiovascular risk prediction score.

In the model including participants with schizophrenia, IL-6 was positively associated with CIMT, and this association remained significant after FDR correction (Table 9).

**Table 9:** Multivariable regression analysis of inflammatory biomarkers with CIMT as the dependent variable.

Model 1	Biomarkers	Standardized β	95% CI	p	FDR-q
Schizophrenia Cytokine Model	lg(IL-1)	0,002	−0,295 – 0,298	0,992	0,992
Schizophrenia Cytokine Model	lg(IL-6)	0,257	0,093 – 0,422	**0,002**	0,015
Schizophrenia Cytokine Model	lg(IL-10)	0,164	−0,014 – 0,342	0,071	0,143
Schizophrenia Cytokine Model	lg(TNF-α)	−0,081	−0,336 – 0,174	0,531	0,797
Schizophrenia Cytokine Model	lg(IFN-γ)	−0,365	−0,695 to −0,034	**0,031**	0,092
Schizophrenia Cytokine Model	lg(NF-κB)	−0,042	−0,315 – 0,232	0,764	0,917

Model 2	Biomarkers	Standardized β	95% CI	p	FDR-q
Overall Cytokine Model	lg(IL-1)	0,065	−0,258 – 0,389	0,690	0,761
Overall Cytokine Model	lg(IL-6)	−0,075	−0,400 – 0,250	0,650	0,761
Overall Cytokine Model	lg(IL-10)	−0,054	−0,406 – 0,297	0,761	0,761
Overall Cytokine Model	lg(TNF-α)	−0,091	−0,331 – 0,150	0,456	0,761
Overall Cytokine Model	lg(IFN-γ)	−0,060	−0,446 – 0,326	0,759	0,761
Overall Cytokine Model	lg(NF-κB)	0,062	−0,195 – 0,320	0,633	0,761

**CI:** confidence interval; **FDR:** false discovery rate; **IL:** interleukin; **TNF-α:** tumor necrosis factor alpha; **IFN-γ:** interferon gamma; **NF-κB:** nuclear factor kappa B.

In the regression models with QRISK3 as the dependent variable, none of the cytokines was significantly associated with QRISK3 in either model (Table 10).

**Table 10:** Multivariable effects of inflammatory biomarkers with QRISK3 as the dependent variable.

Model 1	Biomarkers	Standardized β	95% CI	p	FDR-q
Schizophrenia Cytokine Model	lg(IL-1)	0,069	−0,323 – 0,461	0,729	0,778
Schizophrenia Cytokine Model	lg(IL-6)	0,038	−0,215 – 0,290	0,768	0,778
Schizophrenia Cytokine Model	lg(IL-10)	0,215	0,032 – 0,398	0,022	0,065
Schizophrenia Cytokine Model	lg(TNF-α)	−0,073	−0,287 – 0,140	0,497	0,778
Schizophrenia Cytokine Model	lg(IFN-γ)	−0,390	−0,709 to −0,071	0,017	0,065
Schizophrenia Cytokine Model	lg(NF-κB)	−0,025	−0,202 – 0,152	0,778	0,778

Model 2	Biomarkers	Standardized β	95% CI	p	FDR-q
Overall Cytokine Model	lg(IL-1)	0,131	−0,295 – 0,557	0,543	0,815
Overall Cytokine Model	lg(IL-6)	−0,286	−0,597 – 0,025	0,071	0,427
Overall Cytokine Model	lg(IL-10)	0,003	−0,496 – 0,503	0,990	0,990
Overall Cytokine Model	lg(TNF-α)	−0,083	−0,269 – 0,103	0,378	0,815
Overall Cytokine Model	lg(IFN-γ)	−0,093	−0,546 – 0,359	0,683	0,820
Overall Cytokine Model	lg(NF-κB)	0,076	−0,107 – 0,259	0,414	0,815

**CI:** confidence interval; **FDR:** false discovery rate; **IL:** interleukin; **TNF-α**: tumor necrosis factor alpha; **IFN-γ:** interferon gamma; **NF-κB:** nuclear factor kappa B.

A hierarchical multivariable linear regression analysis was performed to evaluate whether inflammatory biomarkers could independently predict cardiovascular risk after adjustment for traditional cardiovascular risk factors. The CIMT coefficient represents the change in CIMT (mm) per 10-fold increase in the biomarker level, whereas the QRISK3 coefficient represents the change in lg(QRISK3) per 10-fold increase in the biomarker level. Standardized β coefficients are presented to facilitate comparison of effect sizes. After adjustment for the diagnostic group and prespecified cardiovascular and echocardiographic covariates, circulating IL-1, IL-6, IL-10, TNF-α, IFN-γ, and NF-κB levels did not provide a significant additional explanatory contribution to the models for either mean CIMT or QRISK3. These findings do not support an independent and strong association between the inflammatory biomarkers evaluated and the cardiovascular risk measures examined. (Traditional cardiovascular risk factors: LVEF, GLS, E/A ratio, E/e′ ratio, diastolic dysfunction, BMI, waist circumference, systolic blood pressure, and diastolic blood pressure.) (Table 11).

**Table 11:** Regression analysis of the independent predictive value of inflammatory biomarkers for cardiovascular risk after adjustment for traditional cardiovascular risk factors.

Biomarkers	B (95% CI) for CIMT	Standardized β	p	B (95% CI) for lg(QRISK3)	Standardized β	p
lg IL-1	0.010 (−0.100–0.119)	0.027	0.862	−0.088 (−1.980–1.804)	−0.019	0.926
lg IL-6	−0.013 (−0.069–0.043)	−0.059	0.649	−0.836 (−1.864–0.192)	−0.274	0.110
lg IL-10	−0.008 (−0.086–0.070)	−0.031	0.837	−0.476 (−2.048–1.095)	−0.128	0.549
lg TNF-α	−0.013 (−0.072–0.046)	−0.058	0.668	0.201 (−0.543–0.944)	0.072	0.593
lg IFN-γ	0.042 (−0.100–0.184)	0.108	0.557	0.958 (−1.111–3.027)	0.189	0.360
lg NF-κB	−0.021 (−0.076–0.034)	−0.098	0.453	−0.190 (−0.715–0.335)	−0.070	0.474

***CIMT:*** carotid intima–media thickness; ***QRISK3:****cardiovascular risk prediction score****CI:*** confidence interval; ***IL:*** interleukin; ***TNF-α:*** tumor necrosis factor alpha; ***IFN-γ:*** interferon gamma; ***NF-κB:*** nuclear factor kappa B.

To evaluate the potential effect of antipsychotic dose exposure on inflammatory biomarkers, multivariable linear regression analyses were performed exclusively in participants with schizophrenia. The daily chlorpromazine-equivalent dose was entered into the model in 100 mg/day increments. All models were adjusted for age, sex, body mass index, smoking status, and total PANSS score. Because cytokine levels exhibited a skewed distribution, the dependent variables were analysed after log transformation. The standardized beta coefficients for the chlorpromazine-equivalent dose were small across all models, and none of the associations remained statistically significant after FDR correction. In sensitivity analyses restricted to participants with schizophrenia and adjusted for age, sex, BMI, smoking status, and total PANSS score, chlorpromazine-equivalent doses were not associated with peripheral IL-1, IL-6, IL-10, TNF-α, IFN-γ, or NF-κB levels (Table 12).

**Table 12:** Multivariable linear regression analysis of the association between chlorpromazine-equivalent dose and cytokine levels.

Dependent variable	CPZ effect, B per 100 mg	SE	Stand. β	95% CI	Approximate % change per 100 mg CPZ	p	FDR-q	Model R^2^	Adjusted R^2^
lg(IL-1)	−0,012	0,025	−0,059	−0,062 – 0,037	−%1,2	0,625	0,921	0,234	0,132
lg(IL-6)	−0,007	0,032	−0,025	−0,070 – 0,057	−%0,7	0,835	0,921	0,233	0,130
lg(IL-10)	0,023	0,040	0,075	−0,056 – 0,102	+%2,3	0,568	0,921	0,086	−0,037
lg(TNF-α)	−0,034	0,042	−0,104	−0,117 – 0,048	−%3,4	0,410	0,921	0,151	0,037
lg(IFN-γ)	0,002	0,023	0,012	−0,044 – 0,049	+%0,2	0,921	0,921	0,208	0,102
lg(NF-κB)	0,042	0,052	0,101	−0,062 – 0,146	+%4,3	0,421	0,921	0,159	0,046

**CPZ:** chlorpromazine; **SE:** standard error; **β:** standardized beta coefficient; **CI:** confidence interval; **FDR:** false discovery rate; **IL:** interleukin; **TNF-α:** tumor necrosis factor alpha; **IFN-γ:** interferon gamma; **NF-κB:** nuclear factor kappa B.

## 4 Discussion

To the best of our knowledge, our study is the first to concurrently evaluate the increase in cardiovascular risk in schizophrenia in relation to serum cytokines, NF-κB levels, and HLA-DR and TLR expression across monocyte subsets, as well as to investigate the associations between QRISK3 scores and inflammatory markers. In our study, significant differences in immune–inflammatory and cardiovascular markers were identified between the schizophrenia and control groups. In the schizophrenia group, compared with the controls, serum IL-6 and IL-10 levels were elevated, NF-κB and IFN-γ levels were reduced, the proportion of classical monocytes was increased, and the proportions of total and non-classical monocytes were decreased. Additionally, the TLR-2 monocyte percentage, TLR-2 monocyte MFI, TLR-2 classical monocyte MFI, TLR-2 intermediate monocyte MFI, and TLR-2 non-classical monocyte MFI values were significantly higher in the schizophrenia group, whereas the TLR-2 non-classical monocyte percentage was significantly lower.

In the schizophrenia group, the TLR-4 monocyte percentage and the TLR-4 classical monocyte percentage were higher than in the control group, whereas the TLR-4 non-classical monocyte MFI value was lower. Additionally, in the schizophrenia group compared with controls, the HLA-DR monocyte percentage and the HLA-DR intermediate monocyte MFI were higher, whereas the HLA-DR non-classical monocyte percentage was lower. The schizophrenia group also exhibited higher TG levels, QRISK3 scores, CIMT values, and diastolic dysfunction parameters, whereas GLS and the mitral E/A ratio were reduced. The increased IL-6 levels and decreased NF-κB levels were found to be strongly associated with a diagnosis of schizophrenia.

Similarly, in the sensitivity analyses performed exclusively in the patient group and adjusted for age, sex, BMI, smoking status, and PANSS total score, chlorpromazine-equivalent antipsychotic doses were not independently associated with IL-1, IL-6, IL-10, TNF-α, IFN-γ, or NF-κB levels. Among participants with schizophrenia, IL-6 was observed to be positively associated with CIMT, whereas none of the inflammatory biomarkers were observed to be associated with QRISK3.

### 4.1 Inflammatory cytokines in schizophrenia

In our study, IL-6 levels were found to be significantly higher in the schizophrenia group compared to the controls. IL-6, a proinflammatory cytokine, has been reported to be associated with neuroinflammatory processes that may play a role in the pathophysiology of schizophrenia. Elevated IL-6 levels have been reported to be associated with symptom severity and prognosis in schizophrenia at both the central and peripheral levels (Goldsmith et al., 2016). Recent meta-analyses have reported that IL-6 levels are elevated in schizophrenia and other psychiatric disorders compared to healthy controls, and that these levels are influenced by antipsychotic treatment (Y. Zhang et al., 2023; Zhilyaeva et al., 2023). A meta-analysis conducted on individuals with schizophrenia, BPD, and MD found that IL-6 levels decreased following the treatment of acute episodes (Goldsmith et al., 2016). In addition, studies have reported that IL-6 levels are elevated in individuals with ASD compared to controls, and that the combined assessment of IL-6 and serotonin enhances diagnostic sensitivity and specificity in ASD (Yang et al., 2015). The elevated IL-6 levels observed in the schizophrenia group in our study are consistent with previous studies reporting increased peripheral immune activation and inflammatory alterations in schizophrenia. The persistence of elevated IL-6 levels in the schizophrenia group after statistical adjustment for potential confounding variables, including sex, educational level, marital status, smoking, and obesity, may indicate that increased IL-6 levels are associated with immune-inflammatory processes linked to schizophrenia. Nevertheless, it should be taken into consideration that lifestyle factors, stress, dietary habits, metabolic variables, and other environmental factors may also influence the peripheral inflammatory response. Moreover, low-grade chronic inflammation has also been reported in other psychiatric disorders in the literature. Accordingly, it is plausible that the elevation of IL-6 reflects shared inflammatory mechanisms across psychiatric disorders rather than being specific to a single condition (Y. Zhang et al., 2023; Zhilyaeva et al., 2023). In conclusion, further prospective longitudinal studies with larger sample sizes are needed to better understand the biological and clinical significance of IL-6 in schizophrenia. Such studies should include antipsychotic-naive patients, different clinical stages of the disorder, and serial cytokine measurements, while evaluating treatment response and clinical course concurrently. Furthermore, comparative studies involving different psychiatric diagnostic groups may contribute to a more comprehensive evaluation of the disorder-specific and potentially shared characteristics of the IL-6-mediated inflammatory response across psychiatric disorders.

In our study, IL-10 levels were found to be significantly higher in the schizophrenia group compared to the controls. According to the literature, IL-10 has been reported to be one of the principal anti-inflammatory cytokines involved in regulating the balance between proinflammatory and anti-inflammatory cytokines (Saraiva et al., 2020). Findings regarding IL-10 levels in patients with schizophrenia have been inconsistent, and it has been reported that antipsychotic treatment may influence these levels (Beatriz González-Castro et al., 2024). Moreover, studies in this field have reported that IL-10 has a positive correlation with clinical severity in schizophrenia (Xiu et al., 2014). Consistent evidence has demonstrated reductions in gray matter volume and loss of white matter integrity in schizophrenia (van Erp et al., 2018). In schizophrenia, elevated IL-10 levels have been reported to be associated with impaired white matter integrity, which, in turn, may contribute to cognitive deterioration (Najjar and Pearlman, 2015). Therefore, the observed increase in IL-10 may reflect not only a compensatory anti-inflammatory response but also a more complex immunoregulatory pattern that may be associated with the neuroimmune mechanisms reported in the literature.

The observation that recent meta-analyses have reported elevated IL-10 levels in bipolar disorder may suggest that the IL-10-mediated immune response may be associated with immunoregulatory processes that could be shared across different psychiatric disorders rather than being specific to schizophrenia (Y. Zhang et al., 2023). Although elevated IL-10 levels have been considered to potentially reflect a compensatory anti-inflammatory response to proinflammatory activity, the literature also suggests that IL-10 may be associated with alterations in microglial function and white matter integrity under chronic inflammatory conditions (Fu et al., 2014). In this context, the concomitant elevation of IL-6 and IL-10 observed in our study may suggest the presence of concurrent alterations in different components of the peripheral immune response in schizophrenia. However, as the underlying biological mechanisms were not directly evaluated within the scope of our study, further studies are needed to better understand the biological processes underlying this association. In addition, the associations between IL-10 levels and gray and white matter integrity, neuroimaging findings, or neurocognitive function were not directly evaluated in our study. Therefore, further prospective longitudinal studies are needed to examine peripheral IL-10 levels in conjunction with neuroimaging findings, neurocognitive assessments, and gray and white matter integrity, while controlling for the effects of antipsychotic treatment.

In our study, no significant differences were detected between the schizophrenia and control groups in terms of IL-1 and TNF-α levels. According to the current literature, IL-1 and TNF-α have been identified among the principal proinflammatory cytokines associated with schizophrenia. These cytokines have been suggested to exhibit concurrent elevations in both the central and peripheral compartments and may be associated with the neuroimmune response and the pathophysiology of schizophrenia (Goldsmith et al., 2016). Although some studies on schizophrenia and BPD are consistent with our findings, conflicting results have also been reported in the literature (Halstead et al., 2023; Solmi et al., 2021). Nevertheless, the association between IL-1 and TNF-α levels and clinical characteristics has also been reported to vary according to the clinical stage of the disorder and symptom severity (Dunleavy et al., 2022; Patlola et al., 2023). The absence of significant differences in IL-1 and TNF-α levels between the schizophrenia and control groups in our study may suggest that the inflammatory response in chronic or treated patients varies according to the clinical stage of the disorder, treatment duration, and other clinical characteristics. Additionally, the discrepancies may be attributable to methodological variability in sample size, clinical phase, antipsychotic use, and analytical approaches. Given the inconsistent findings reported in the current literature, further prospective longitudinal studies that take into account sample characteristics, different clinical stages of the disorder, antipsychotic treatment status, and serial cytokine measurements are needed to better clarify the biological significance of IL-1 and TNF-α in schizophrenia. Furthermore, larger studies incorporating neuropathological tissue analyses are warranted to more comprehensively validate these findings and to further elucidate the underlying biological mechanisms.

In our study, NF-κB levels were found to be significantly lower in the schizophrenia group than in the controls. NF-κB has been reported to be associated with the regulation of neuroinflammatory processes and synaptic plasticity in schizophrenia (Najjar and Pearlman, 2015). Experimental studies have reported that NF-κB activation may lead to increased levels of proinflammatory cytokines. This increase has been suggested to enhance glutamate release, reduce its reuptake, and contribute to elevated synaptic glutamate levels (Zou and Crews, 2005). Furthermore, the literature suggests that NF-κB may be associated with inflammatory processes in MD and neuroprogression in BD, and may represent one of the immune pathways potentially involved in neurodevelopmental processes in ASD and neurodegenerative mechanisms in Alzheimer's disease (Heneka et al., 2015; A. H. Miller and Raison, 2016).

Postmortem brain tissue studies and some investigations conducted in patients with chronic schizophrenia have reported suppression of the NF-κB pathway in various brain regions (Roussos et al., 2013). In contrast, the literature also includes studies reporting increased NF-κB activity in schizophrenia. However, a substantial proportion of these studies were conducted in peripheral mononuclear cells, during acute exacerbations, or in experimental models (Song et al., 2009; Volk et al., 2019). These differences may be attributable to the biological specimen examined (serum, PBMCs, or brain tissue), the stage of the disorder, the treatments used, and the specific NF-κB subunits evaluated. Therefore, when considered together with the existing literature, our findings may suggest that the discrepant findings regarding NF-κB levels in schizophrenia may be associated with various factors, including the stage of the disorder, the biological material sampled, and clinical characteristics. Furthermore, studies have indicated that lithium may induce significant changes in the NF-κB signaling pathway, whereas the effects of different antipsychotics on NF-κB activity have been reported to be heterogeneous (Gao et al., 2024; Sun et al., 2024). Experimental and clinical studies have shown that some antipsychotics may suppress microglial activation and NF-κB signaling (M. L. Chen et al., 2011). Nevertheless, although IL-6 and IL-10 levels were elevated in our study, NF-κB levels were reduced, which may appear contradictory at first glance. Although NF-κB is one of the principal regulators of inflammatory cytokine production, its activity is known to vary over time during chronic inflammatory processes (Lawrence, 2009). Although NF-κB activation may trigger cytokine production during the acute phase, NF-κB signaling has been shown to be suppressed in systems exposed to prolonged inflammatory stimuli through IκB proteins, A20 (TNFAIP3), and other negative feedback mechanisms (Lawrence, 2009; Verstrepen et al., 2010). In this context, while the elevated serum cytokine levels may reflect previous or ongoing inflammatory activation, the reduced NF-κB levels may reflect a compensatory downregulation in response to chronic inflammation. These possibilities should not be overlooked, particularly in our sample, which consisted of patients in remission who had been receiving long-term antipsychotic treatment. However, these mechanisms were not directly evaluated in our study. In our study, hierarchical logistic regression analysis demonstrated a negative association between NF-κB levels and a diagnosis of schizophrenia. In other words, lower NF-κB levels were associated with a higher likelihood of belonging to the schizophrenia group. This finding may suggest that NF-κB may not serve as a standalone biomarker that linearly reflects the severity of inflammation, but rather may reflect a more complex pattern of immune regulation shaped by the chronic course of the disorder, long-term antipsychotic treatment, and negative feedback mechanisms. Furthermore, studies in certain neurodegenerative disorders have reported that reductions in specific components of NF-κB signaling may contribute to disease pathophysiology. In particular, reduced expression or functional loss of the neuroprotective c-Rel subunit has been reported to be associated with dopaminergic neuronal loss in experimental Parkinson's disease, whereas certain genetic alterations that attenuate NF-κB signaling have been suggested to contribute to disease development in Alzheimer's disease (Baiguera et al., 2012; Kaltschmidt et al., 2024). These findings may suggest that reduced NF-κB activity may not always indicate suppression of the inflammatory response, but may also reflect disruption of neuronal homeostasis and neuroprotective mechanisms in certain disease contexts. Similarly, neuroprogressive processes, in addition to neurodevelopmental mechanisms, have been suggested to contribute to the course of schizophrenia (Reckziegel et al., 2022). Therefore, the reduced serum NF-κB levels observed in our study may represent not only a peripheral reflection of compensatory downregulation in response to chronic inflammation, but also a peripheral reflection of potential alterations in neuroprotective NF-κB signaling. However, NF-κB levels were measured in serum samples in our study, and this measurement does not directly reflect the intracellular activation status or nuclear translocation of NF-κB. Therefore, the findings should be interpreted in terms of circulating NF-κB levels rather than the functional activity of NF-κB signaling.

In our study, the intracellular activation of NF-κB signaling, subunit-specific regulation, and its downstream biological effects were not directly evaluated. Furthermore, owing to the cross-sectional design of our study and the use of single time-point serum measurements, our findings do not allow us to determine conclusively whether the reduced NF-κB levels observed are a feature specific to schizophrenia. Therefore, interpretations regarding the effects of glutamatergic and dopaminergic pathways, epigenetic regulation, negative feedback mechanisms, and antipsychotic treatment on NF-κB should be regarded not as definitive conclusions based on our findings, but rather as potential biological explanations in light of the existing literature. Therefore, prospective longitudinal studies with larger sample sizes are needed to comprehensively evaluate NF-κB subunits, intracellular activation patterns, central and peripheral immune responses, glutamatergic and dopaminergic pathways, the effects of antipsychotic treatment, and disease stage, while also incorporating repeated NF-κB measurements throughout the day.

Serum IFN-γ levels were observed to be lower in the schizophrenia group compared to the controls. According to the literature, IFN-γ may be associated with microglial activation, neuroinflammation, and the kynurenine pathway. Through these mechanisms, it has been suggested to influence NMDA receptor function and dopaminergic signaling and, consequently, may be associated with the pathophysiology and clinical characteristics of schizophrenia (Schwarcz and Stone, 2017). Alterations in IFN-γ levels have also been reported not to be specific to schizophrenia and may be associated with a range of neuropsychiatric disorders (Najjar and Pearlman, 2015). Studies on schizophrenia have reported that IFN-γ levels may vary from the acute to the chronic phase of the disorder (Halstead et al., 2023). Findings in individuals diagnosed with BPD and MD appear to be consistent with our results, and the literature also reports that psychotropic treatments can influence IFN-γ levels (Damri and Agam, 2024). The reduced IFN-γ levels observed in patients with schizophrenia in remission in our study are consistent with previous reports describing suppressed IFN-γ levels during the chronic phase of the disorder. In contrast to the transient elevations reported during the acute phase, this reduction may be consistent with the pattern of immune suppression described in the literature for the chronic stage of schizophrenia (Goldsmith et al., 2016). Therefore, our findings may indicate that reduced IFN-γ levels are associated with the immune alterations reported during the chronic phase of schizophrenia. However, as these mechanisms were not directly evaluated in our study, further prospective longitudinal studies with larger sample sizes are needed to more comprehensively clarify the potential association between reduced IFN-γ levels and these biological processes. Such studies should include antipsychotic-naive or first-episode patients, encompass different clinical stages of the disorder, and assess serum IFN-γ levels together with central biomarkers derived from cerebrospinal fluid, neuroimaging, and appropriate neuropathological tissue samples.

### 4.2 Toll-like receptors and HLA expression in monocyte subtypes in schizophrenia

In our study, individuals with schizophrenia exhibited lower total and non-classical monocyte proportions compared to healthy controls, whereas classical monocyte proportions were increased. No significant difference was observed in intermediate monocyte proportions. Monocytes and macrophages are key components of the innate immune system and play a critical role in initiating and regulating inflammatory responses (Dudeck et al., 2025a, Dudeck et al., 2025b). Nevertheless, the inflammatory response is not regulated solely by monocytes; neutrophils, lymphocytes, dendritic cells, and other immune cells also play important roles in this process (Liu et al., 2017). In this context, large-scale meta-analyses published in recent years have shown that peripheral neutrophil counts are increased in patients with schizophrenia, whereas findings regarding different lymphocyte subsets and other immune cells have been variable depending on disease stage and treatment status (Dudeck et al., 2025a, Dudeck et al., 2025b). However, monocytes were selected for the present study because studies evaluating innate immune system activation in schizophrenia have suggested that monocytes may play a central role in the interaction between the peripheral immune response and the central nervous system (Khandaker et al., 2015). Circulating monocytes can interact with the blood–brain barrier, secrete cytokines capable of influencing microglial activation, and are considered an appropriate cellular model for assessing the peripheral manifestations of neuroinflammatory processes (Takahashi et al., 2016). Indeed, recent studies have reported that the most consistent alterations observed in the peripheral innate immune system in schizophrenia involve monocyte counts, the distribution of monocyte subsets, and monocyte function (Dudeck et al., 2025a, Dudeck et al., 2025b). In addition, classical, intermediate, and non-classical monocyte subsets are known to possess distinct functional characteristics with respect to inflammatory responses, endothelial interactions, tissue migration, and antigen presentation (Kapellos et al., 2019). Therefore, examining monocyte subsets rather than total monocyte counts may provide more detailed information regarding the nature of the immune response. Furthermore, monocytes are among the principal peripheral immune cells that express high levels of key regulators of the innate immune response, including TLR-2, TLR-4, and HLA-DR (Simpson et al., 2009). Because of these characteristics, monocytes are widely regarded as one of the cell populations used to evaluate innate immune system activation and alterations in antigen-presenting capacity. This approach does not preclude the importance of other immune cells, such as neutrophils, lymphocytes, or dendritic cells, in schizophrenia. However, because the aim of the present study was to evaluate the monocyte-mediated components of the innate immune response, changes in TLR-2, TLR-4, and HLA-DR expression on monocytes, and the potential associations of these immunological characteristics with cardiovascular risk markers, the analyses were specifically focused on monocyte subsets. Therefore, the evaluation of monocyte subsets may provide an approach that could contribute to a better understanding of the biological basis of these disorders and facilitate a more detailed assessment of the alterations observed in the peripheral immune system in future studies.

Recent studies have indicated that meaningful alterations in monocyte function and subset distribution may occur not only in schizophrenia and BPD but also in other neuropsychiatric conditions such as AD and ASD (Goines and Ashwood, 2013; Huang et al., 2024). These findings may suggest that neuropsychiatric disorders may share a common immunoinflammatory basis and may underscore the importance of considering the immunological effects of clinical treatments (B. J. Miller et al., 2011). Clozapine, which is widely used in treatment-resistant schizophrenia, may cause agranulocytosis. Clozapine-induced agranulocytosis is thought to result from reactive clozapine metabolites triggering apoptosis in neutrophil precursor cells and enhancing immune-mediated neutrophil destruction. These findings may suggest that immune system function may be important both for understanding the immunological characteristics of schizophrenia and for evaluating the immunological effects of antipsychotic treatments (Ng et al., 2014). Therefore, alterations in monocyte and macrophage function may be important not only for understanding the immunological characteristics of the disorder but also for evaluating the effects of antipsychotic treatments on the immune system.

A recent study reported that the proportion of non-classical monocytes was lower in the schizophrenia group compared to controls, whereas no significant differences were observed between the groups in classical, intermediate, or total monocyte proportions (S. Chen et al., 2022). Similar patterns were observed in ASD and MD. The authors emphasised that the increase in classical monocyte proportions may be attributable to enhanced monopoiesis in the bone marrow, whereas the reduction in non-classical monocytes was interpreted as a consequence of their migration into tissues, particularly the intestinal epithelium and endothelium, to perform repair functions (Daray et al., 2024). Nevertheless, because monocyte migration, endothelial repair capacity, and tissue-level functional outcomes were not directly evaluated in the present study, further studies incorporating analyses that directly assess monocyte function, endothelial-level investigations, and longitudinal follow-up data are needed to better understand these processes.

The distribution of monocyte subsets observed in our schizophrenia cohort was partially consistent with the existing literature, although some differences were also identified. The variability in findings regarding monocyte subsets in psychiatric disorders may be related to differences in sample characteristics, clinical stage, pharmacotherapy history, and methodological variations in flow cytometry panels. Moreover, the dynamic nature of monocyte subsets, including the short lifespan of classical monocytes and the tissue-migratory propensity of non-classical subsets, may further contribute to this biological heterogeneity (S. Chen et al., 2022). The profile observed in our schizophrenia sample parallels the “classical↑/non-classical↓” pattern reported in individuals with autism, yet it diverges from the increased non-classical proportions described in depressive cohorts (Daray et al., 2024). Therefore, the alterations in monocyte subsets observed in psychiatric disorders may be influenced by both disorder-specific biological processes and treatment-related immune modulation. In future studies, careful control of disease stage, treatment duration, diet, smoking status, and metabolic variables, together with the standardisation of flow cytometry analyses, may help reduce methodological heterogeneity across studies. Such approaches may facilitate a better understanding of whether the observed differences in immune cell subsets are associated with disorder-specific biological processes, treatment-related immune modulation, or methodological variation.

In our study, the schizophrenia group exhibited higher TLR-2 monocyte percentages, alongside increased TLR-2 monocyte MFI and elevated MFI values in classical, intermediate, and non-classical monocyte subsets compared with controls, while the TLR-2 non-classical monocyte percentage was lower. Additionally, TLR-4 monocyte and TLR-4 classical monocyte percentages were elevated in the schizophrenia group, whereas the TLR-4 non-classical monocyte MFI value was reduced. The literature has reported that TLR-2 and TLR-4 are receptors involved in the regulation of the innate immune response (Kawasaki and Kawai, 2014). Experimental studies have reported that TLR-2 and TLR-4 activation is associated with microglial inflammation and the production of proinflammatory cytokines, and that these processes may influence synaptic remodeling (Kéri et al., 2017). Recent studies have reported inconsistent findings regarding TLR-2 and TLR-4 levels, and psychotropic medications have been shown to induce changes in the expression of these receptors (Jameie et al., 2025).

Studies on schizophrenia have suggested that excessive acceleration or dysregulation of physiological synaptic pruning during adolescence and early adulthood may be associated with the neurodevelopmental processes underlying the disorder (Howes and Shatalina, 2022). Similarly, experimental studies have reported that TLR-2- and TLR-4-mediated microglial activation may be associated with synaptic pruning processes and may contribute to synaptic loss through increased phagocytosis (Ramirez et al., 2026). In this context, TLR-mediated neuroimmune processes have been reported to be associated with reduced synaptic density in the prefrontal cortex and hippocampus, and these alterations may be associated with clinical severity in schizophrenia and bipolar disorder (Rosenblat and McIntyre, 2016). Furthermore, the literature suggests that TLR-2 and TLR-4 may be associated with neurodevelopmental and neurodegenerative processes. In this context, the literature suggests that these processes may be associated with disruptions in neurodevelopmental circuit formation in schizophrenia and ASD, whereas they may be associated with progressive neurodegenerative mechanisms in BD, MD, and AD (Heneka et al., 2015). Therefore, the TLR-2 and TLR-4 findings from our study may be interpreted within a hypothesis-generating framework with respect to neuroimmune mechanisms that may be associated with early synaptic circuit formation and later neuronal integrity in psychiatric disorders. Nevertheless, synaptic pruning, microglial activation, neuroimaging findings, and neurodegenerative processes were not directly evaluated in our study. Therefore, further prospective longitudinal studies including antipsychotic-naïve patients, different clinical stages of the disorder, and different psychiatric diagnostic groups are needed to better understand the potential role of TLR-2- and TLR-4-mediated neuroimmune mechanisms in schizophrenia and other psychiatric disorders. Furthermore, comparative studies integrating peripheral immune markers with neuroimaging findings, neurocognitive assessments, biomarkers reflecting synaptic integrity, and, where appropriate, cerebrospinal fluid or tissue-based analyses may contribute to a more comprehensive evaluation of the relationship between peripheral immune alterations and central neuroimmune processes. Comparative studies conducted across different psychiatric disorders may contribute to a better understanding of the disorder-specific and shared biological patterns associated with TLR-2- and TLR-4-mediated neuroimmune mechanisms.

Findings related to the TLR system also reveal immune response differences in other neuropsychiatric disorders. A recent study reported that individuals with type I BPD exhibited higher baseline TLR-2 monocyte proportions compared with controls, and that this increase became more pronounced following monocyte stimulation (Wieck et al., 2016). In a study investigating the aetiology of ASD, pre-pregnancy infections were found to be associated with increased placental TLR-2 monocyte expression; this elevation was reported to heighten the risk of low birth weight, impaired fetal development, and subsequent neurodevelopmental disorders (Liao et al., 2018). Our findings may indicate that TLR-2 and TLR-4 expression in the schizophrenia group exhibits distinct patterns not only at the level of total monocytes but also across monocyte subsets. In patients with schizophrenia, the concurrent increase in MFI values in classical and intermediate monocytes, together with the simultaneous increase in MFI values within the non-classical monocyte subset despite a reduction in the proportion of these cells, may suggest that the immune characteristics of monocyte subsets are affected differently. These findings may indicate that the immunological differences among monocyte subsets cannot be explained solely by changes in receptor expression. However, further prospective longitudinal studies directly evaluating monocyte function and including antipsychotic-naïve patients as well as different clinical stages of the disorder are needed to clarify the functional mechanisms underlying these differences. The increased proportion of classical monocytes and the reduced MFI values in non-classical monocytes observed within the TLR-4 axis were partially consistent with immune patterns that have been associated with chronic low-grade inflammation and immunosenescence in the literature (Kapellos et al., 2019; Müller et al., 2012). However, because functional tests or specific markers of immunosenescence were not directly evaluated in our study, these findings should not be interpreted as direct evidence of immunosenescence but rather should be regarded as hypothesis-generating findings in the context of immune patterns that have been associated with immunosenescence in the literature.

The heterogeneity in TLR-2 and TLR-4 expression observed across monocyte subsets may represent one of the possible explanations for the inconsistencies reported in previous studies that focused solely on total monocyte levels. These findings may suggest that evaluating TLR pathways not only at the level of total monocytes but also across monocyte subsets may be useful not only in schizophrenia but also in other neuropsychiatric disorders in which inflammatory mechanisms may play a role. Furthermore, because psychotropic medications have been reported to influence TLR expression, it may be important for future studies to evaluate the potential effects of antipsychotic treatment on monocyte subsets separately. Overall, our findings may suggest that approaches focusing on specific cellular components of the innate immune system and monocyte subsets may contribute to a more detailed evaluation of the immune alterations observed in schizophrenia.

In our study, the schizophrenia group exhibited higher monocyte HLA-DR proportions and elevated intermediate HLA-DR monocyte MFI levels compared with the controls, whereas the proportion of non-classical HLA-DR monocytes was reduced. In contrast, no significant differences were observed in classical monocyte HLA-DR proportions or in overall monocyte HLA-DR MFI values. HLA-DR is a key surface marker on monocytes and macrophages that reflects both cellular activation and antigen-presenting capacity (Van Kesteren et al., 2017). Alterations in HLA-DR levels in psychiatric disorders are considered an important biomarker of immune activation. Studies have suggested that increased HLA-DR expression on monocytes in schizophrenia and bipolar disorder may be associated with microglial activation, proinflammatory cytokine responses, and synaptic pruning processes (Van Kesteren et al., 2017). Similarly, the literature suggests that microglial activation in schizophrenia may be associated with increased HLA-DR-mediated inflammatory responses, kynurenine pathway activity, and dopaminergic dysregulation through NMDA receptor inhibition (Müller, 2008). In addition, increased HLA-DR activity in monocytes has been reported in ASD and AD, a finding linked to disrupted early neuronal network organisation and the acceleration of neurodegenerative mechanisms in later stages (Vargas et al., 2005; R. Zhang et al., 2013). Nevertheless, because microglial activation, kynurenine pathway activity, synaptic pruning, and neurodegenerative processes were not directly evaluated in our study, further prospective longitudinal studies integrating peripheral immune markers with neuroimaging findings, neurocognitive assessments, and different clinical stages of the disorder are needed to better understand the potential association between alterations in HLA-DR expression and these mechanisms.

The literature suggests that HLA-DR may represent an important immunological marker for evaluating immune activation across various psychiatric disorder (Laricchiuta et al., 2024). Studies in schizophrenia and BPD have demonstrated increased monocyte HLA-DR proportions relative to healthy controls, with evidence indicating that this elevation tends to normalise following treatment. Found that these cells also exhibit a blunted response to stimulation compared with those of healthy controls. They interpreted the diminished inducibility accompanying elevated basal activity as a marker of immune dysregulation and possible immune exhaustion in schizophrenia (Nguyen et al., 2023).

Overall, our findings appear to be consistent with those reported in the existing literature. Our findings may indicate that alterations in HLA-DR expression are present across monocyte subsets in schizophrenia. In particular, the increased HLA-DR expression observed in the intermediate monocyte subset may reflect an immune phenotype that may be associated with differences in peripheral immune activation. In contrast, the reduced proportion of HLA-DR-positive non-classical monocytes in the schizophrenia group may indicate an alteration in the distribution of this monocyte subset, which has been reported to be associated with the maintenance of vascular integrity, endothelial repair, and the regulation of inflammatory responses (Tahir and Steffens, 2021). However, because the functional properties of these cells and their endothelial repair capacity were not directly evaluated in our study, further prospective longitudinal studies evaluating monocyte function, endothelial function, and vascular markers together are needed to better understand these processes. Furthermore, the differences in HLA-DR expression observed across monocyte subsets may indicate that HLA-DR expression may exhibit a heterogeneous distribution among monocyte subsets and that immune activation may display distinct characteristics at the subset level. Therefore, our findings may suggest that peripheral immune alterations involving monocytes and monocyte subsets in schizophrenia may exhibit a heterogeneous pattern. Validation of our findings in larger cohorts, together with support from prospective longitudinal studies including antipsychotic-naïve patients, different clinical stages of the disorder, and functional immune assessments, may contribute to a more comprehensive evaluation of the biological and clinical significance of alterations in HLA-DR expression at the level of monocyte subsets, their relationship with peripheral immune activation, and their potential clinical implications.

### 4.3 Relationship between QRISK3, cardiometabolic parameters, and inflammatory cytokines in schizophrenia

In our study, evaluation of the lipid profile revealed that the mean TG levels were significantly higher in the schizophrenia group compared to the controls, whereas no significant differences were observed for the other parameters. In the cardiovascular assessments, the schizophrenia group exhibited higher QRISK3 scores, greater mean CIMT, and more frequent diastolic dysfunction than the controls, whereas GLS and the mitral valve E/A ratio were lower. In contrast, no significant differences were observed between the groups in terms of the mitral valve E/E ratio or FMD levels.

TG levels were found to be higher in the schizophrenia group than in the controls. Dyslipidaemia is a common metabolic disturbance in patients with schizophrenia and is largely associated with lifestyle factors and the adverse metabolic effects of antipsychotic treatment (Baller et al., 2015). Meta-analyses have shown that TG levels are significantly elevated in individuals with schizophrenia compared with controls, and that this increase is positively correlated with clinical severity (Ali et al., 2024; Jirakran et al., 2025). Moreover, several studies have reported that TG levels are influenced by psychotropic treatment (Li et al., 2024). The higher TG levels observed in the schizophrenia group in our study are consistent with the dyslipidaemia commonly reported in schizophrenia and suggest that the biological and behavioural processes accompanying the disorder may influence lipid metabolism. Given that elevated TG levels increase atherogenic risk, this finding may indicate an additional cardiovascular risk in individuals with schizophrenia.

Since the use of psychotropic medications is widespread in schizophrenia, the extent to which the observed increase in TG levels was attributable to antipsychotic treatment could not be clearly determined. To more accurately evaluate the effects of medications on the lipid profile, advanced studies including individuals who are medication-free and without comorbidities are needed. Considering that an increase in TG levels may arise from the interaction of genetic, environmental, and pharmacological factors, additional studies based on controlled and comprehensive evaluations are required.

In our study, the mean CIMT levels were found to be increased in the schizophrenia group compared with the controls. CIMT is an early indicator of atherosclerosis and reflects subclinical alterations in the arterial wall (Bauer et al., 2012). Increased CIMT has been reported to be associated with cognitive impairment and various CVD in schizophrenia (Tsai et al., 2022). Recent meta-analyses in the fields of schizophrenia and MD have been consistent with our findings (Kapıcı et al., 2024; Saleh et al., 2024). Our finding is consistent with the peripheral inflammatory and subclinical vascular alterations reported in schizophrenia and may suggest a potential association with atherosclerotic changes. CIMT is a marker that reflects cardiovascular risk along a continuous spectrum, and even small increases have been reported to be associated with an increased risk of future cardiovascular events at the population level (van den Oord et al., 2013). In this context, the clinical significance of our findings may be interpreted not as indicating the presence of established atherosclerotic disease, but rather as representing a marker that may contribute to the evaluation of early subclinical vascular alterations and findings that may be associated with an increased cardiovascular risk in patients with schizophrenia.

Chronic inflammation, oxidative stress, metabolic disturbances, and the antipsychotic-related increase in CVD risk observed in schizophrenia may be among the potential contributors to these vascular alterations (J. Chen et al., 2025). Therefore, our findings may suggest the importance of monitoring early vascular changes in schizophrenia. From a clinical perspective, CIMT measurement may be regarded as an adjunctive parameter that supports cardiovascular risk assessment rather than as a standalone diagnostic test. Furthermore, the reported association between increased CIMT and cognitive impairment may suggest that this parameter has clinical relevance not only in terms of cardiovascular risk but also with respect to neuropsychiatric outcomes (Tsai et al., 2022). Nevertheless, because of the cross-sectional design of our study, the absence of cognitive assessments, and the lack of evaluation of biomarkers directly reflecting central neuroinflammation, this relationship could not be directly established. Future prospective studies with larger sample sizes, including drug-naïve or first-episode patients and integrating cognitive assessments with neuroinflammatory biomarkers obtained from cerebrospinal fluid, endothelial, and tissue samples, are needed.

In this study, the mitral valve E/A ratio was found to be lower and the rate of diastolic dysfunction higher in the schizophrenia group. Diastolic dysfunction refers to an impairment in the relaxation and filling phase of the heart. Studies conducted in the general population have suggested that diastolic dysfunction may be associated with various cardiovascular events and heart failure over the long term (Ladeiras-Lopes et al., 2019). In recent years, growing evidence has suggested that diastolic dysfunction may be associated not only with classical CVD but also with systemic inflammation, metabolic disturbances, and psychiatric disorders (Yaqub et al., 2025). The literature reports that the reduced mitral valve E/A ratio and increased frequency of diastolic dysfunction observed in schizophrenia and BPD may be associated with neuroanatomical and neurodegenerative changes (P. H. Chen et al., 2025).

However, some studies have indicated that the low E/A ratio may also be attributable to antipsychotic treatment (Curto et al., 2015). In our study, the impairment in diastolic function in the schizophrenia group was found to parallel the reduction observed in the mitral valve E/A ratio. This finding may suggest that alterations in the relaxation and filling phases of the heart may be present in patients with schizophrenia. Nevertheless, the clinical significance of these alterations and their potential relationship with long-term cardiovascular outcomes could not be evaluated within the scope of our study. From a clinical perspective, these findings may reflect early alterations in diastolic function that may occur before the development of clinically overt cardiac dysfunction, rather than established diastolic heart failure.

Our findings are consistent with the current literature in this field. In this context, non-invasive and sensitive cardiac parameters such as the E/A ratio may be regarded as adjunctive echocardiographic markers that support the early evaluation of cardiovascular involvement in patients with schizophrenia. Nevertheless, the relationship between this finding and neurovascular or neuroanatomical alterations in patients with schizophrenia could not be clearly established in our study. Therefore, comprehensive studies evaluating endothelial function not only at the peripheral level but also at the vascular tissue level, while simultaneously examining the effects of antipsychotic treatment, biomarkers associated with neuroimmune processes, and accompanying vascular and structural–functional brain alterations, are needed. Furthermore, long-term prospective longitudinal studies including antipsychotic-naïve or first-episode patients are needed to determine whether the early alterations in diastolic function observed in patients with schizophrenia are associated with the subsequent development of clinically significant diastolic dysfunction, heart failure, or other cardiovascular events.

In this study, mean GLS values were found to be lower in the schizophrenia group. Lower GLS may indicate subclinical left ventricular dysfunction, even when the ejection fraction is normal, and may reflect early myocardial effects of chronic inflammation, oxidative stress, and metabolic disturbances accompanying schizophrenia (Correll et al., 2017). Nevertheless, the reduced GLS value observed in the schizophrenia group may not, by itself, indicate the presence of clinically overt heart failure or symptomatic cardiac dysfunction. Studies have suggested that GLS is a sensitive echocardiographic parameter that may detect myocardial dysfunction at an early stage, even in individuals with preserved ejection fraction (Potter and Marwick, 2018). Therefore, these findings may suggest subclinical myocardial involvement rather than established clinical cardiac disease, and closer cardiovascular follow-up may be beneficial in these patients. Therefore, GLS may be regarded as an adjunctive echocardiographic parameter that may contribute to the evaluation of subclinical alterations in cardiac function in patients with schizophrenia.

There is limited research examining the relationship between schizophrenia and GLS. Recent studies have reported that GLS values are lower in individuals with schizophrenia compared with controls, that reduced GLS is associated with clinical severity, and that psychotropic medications can influence GLS levels (P. H. Chen et al., 2025). Therefore, our findings may suggest that the cardiac alterations observed in schizophrenia are associated not only with traditional cardiovascular risk factors but also with other biological and clinical processes. Although certain antipsychotic medications are known to exert varying effects on cardiac load, our study did not evaluate the relationship between GLS and treatment duration or type of medication. Therefore, comprehensive longitudinal studies that incorporate treatment-related variables are needed to elucidate the mechanisms underlying the observed alterations. In addition, our study did not evaluate whether the reduction in GLS values exceeded established clinical threshold values or whether it was associated with long-term cardiovascular outcomes. Furthermore, because of the cross-sectional design of our study, the temporal and causal relationships between reduced GLS values and the biological processes accompanying schizophrenia or subsequent cardiovascular outcomes could not be evaluated. Therefore, although our findings may indicate the presence of subclinical cardiac alterations in patients with schizophrenia, further prospective longitudinal studies that take into account antipsychotic treatment, disease duration, and other cardiovascular risk factors are needed to better define the clinical and prognostic significance of these alterations.

In our study, the mean QRISK3 score was higher in the schizophrenia group compared with the controls, and this score was found to be positively correlated with CIMT and negatively correlated with IL-6 and TNF-α levels. In addition, hierarchical regression analyses using QRISK and CIMT as dependent variables showed that IL-6 was positively associated with CIMT, whereas none of the inflammatory markers was associated with QRISK.

QRISK3 is a multivariable risk estimation algorithm designed to predict the likelihood of developing cardiovascular disease within 10 years in individuals with psychiatric diagnoses. In addition to classical cardiovascular risk factors, this algorithm incorporates psychiatric components such as depression, schizophrenia, BPD, and antipsychotic medication use into the model (Hippisley-Cox et al., 2017). The higher QRISK3 scores observed in the schizophrenia group in our study may be considered an expected finding. This is because the algorithm itself incorporates both a diagnosis of schizophrenia and antipsychotic use as components of cardiovascular risk estimation. Therefore, the difference observed between the groups may reflect not only biological differences but also the structural characteristics of the algorithm itself. Therefore, the higher QRISK3 scores observed in the schizophrenia group in our study may be interpreted not as independent evidence of an increased cardiovascular risk in schizophrenia, but rather as a finding indicating that a validated risk algorithm performed as expected in our study population. Although studies examining the relationship between schizophrenia and QRISK3 are limited, existing research has reported that individuals with schizophrenia spectrum disorders exhibit higher QRISK3 scores compared with controls (Örüm et al., 2024).

Most studies examining the relationship between QRISK3 scores, CIMT, and cytokine levels have focused primarily on rheumatologic diseases. In inflammatory disorders, such as rheumatoid arthritis, systemic lupus erythematosus, and inflammatory bowel disease, QRISK3 has been reported to show a positive correlation with CIMT and carotid plaque (Gerasimova et al., 2023). Similarly, in various inflammatory diseases, mean QRISK3 scores have been reported to show a positive association with inflammatory cytokines such as CRP and IL-6 (Espinosa-Bautista et al., 2025). Nevertheless, most of these studies have been conducted in chronic inflammatory diseases, and to the best of our knowledge, studies evaluating the relationship between QRISK3 scores and inflammatory markers in patients with schizophrenia remain limited.

The positive correlation observed between QRISK3 scores and CIMT in our study may suggest a relationship between the overall cardiovascular risk profile and subclinical vascular alterations in patients with schizophrenia. Nevertheless, because of the cross-sectional design of our study, the direction of this relationship and any causal inference could not be established. Furthermore, QRISK3 is not a single biological marker but a composite risk algorithm incorporating multiple clinical variables. Therefore, the observed correlation with CIMT may be interpreted as reflecting the relationship between overall clinical risk burden and vascular alterations rather than a specific biological mechanism.

Our adjusted analyses may suggest that inflammatory and cardiovascular risk markers vary concurrently in schizophrenia; however, cytokines do not appear to independently account for these cardiovascular markers to a substantial extent. This finding may indicate that the relationship between cardiovascular risk and systemic inflammatory markers in schizophrenia is more complex and that the present findings require further confirmation in future studies. These regression findings may reflect the cross-sectional design of our study. Nevertheless, it should also be taken into consideration that the findings obtained from the regression analyses may have been influenced by the cross-sectional design of our study.

One possible explanation for the lack of an independent association between QRISK3 and peripheral inflammatory markers in our study may be that QRISK3 is a composite cardiovascular risk prediction algorithm reflecting the cumulative effects of multiple clinical cardiovascular risk factors, whereas peripheral cytokine levels are more dynamic biomarkers that reflect an individual's inflammatory status at the time of measurement (Del Giudice and Gangestad, 2018; Hippisley-Cox et al., 2017). Therefore, it may be expected that the alterations observed in peripheral inflammatory markers do not necessarily parallel the long-term cardiovascular risk represented by QRISK3. Another possible explanation for this finding in our study may be that peripheral cytokine levels vary according to disease stage, symptom severity, and clinical stability (Goldsmith et al., 2016). The inclusion of only patients in remission in our study may have contributed to this finding. In addition, because of the cross-sectional design of our study, inflammatory markers were assessed at a single time point only, and the temporal changes in cytokine levels could not be evaluated together with the longitudinal course of cardiovascular risk markers. This methodological limitation may have influenced the interpretation of our findings and contributed to the observed results. In addition, the multivariable nature of the QRISK3 algorithm should be taken into consideration when interpreting QRISK3 scores. Therefore, the associations observed between QRISK3 and inflammatory markers in our study do not demonstrate direct biological causality but may reflect potential relationships between the overall clinical cardiovascular risk burden and the peripheral inflammatory profile.

In our study, the relationship between cardiovascular risk and inflammatory markers in schizophrenia could not be clearly established. Therefore, further prospective longitudinal studies with larger sample sizes are needed to confirm the association between QRISK3 and inflammatory markers and to provide a more comprehensive evaluation of the factors that may influence this relationship. Such studies should include antipsychotic-naïve patients, assess different stages of the disorder, incorporate repeated measurements of inflammatory markers throughout the day, carefully control for the clinical variables included in the QRISK3 algorithm, and evaluate alternative cardiovascular risk markers.

### 4.4 Limitations

Among the strengths of our study is the integrated evaluation of clinical, biochemical, immunological, and cardiological indicators. Our study included only patients diagnosed with schizophrenia, while other psychotic disorders were systematically excluded. The participants in both groups were comparable in terms of age and sex, and potential confounders such as age, BMI, sex, education level, marital status, socioeconomic status, and smoking were controlled for, with adjustments made for multiple comparisons. Hierarchical logistic regression analyses were employed to identify immune–inflammatory markers predictive of schizophrenia diagnosis. Because a large number of inflammatory, flow cytometric, and cardiovascular variables were examined in this study, efforts were made to minimise the potential increase in the risk of Type I error. Therefore, Bonferroni correction was applied within biologically related outcome groups. In our study, multiple regression analyses were performed to examine the relationship between inflammatory markers and cardiovascular risk in schizophrenia, and efforts were made to adjust the inflammatory markers for traditional cardiovascular risk measures. A post hoc power analysis was also attempted to assess whether the sample size was adequate in our study. Therefore, although a post hoc power analysis was conducted, the sample size was not determined on the basis of a predefined statistical power target.

However, this study also has several limitations. Due to the cross-sectional design, the findings can be interpreted at an associative rather than a causal level. The fact that all patients in our study were receiving antipsychotic treatment represents an important potential confounding factor because of the known effects of these medications on immunological, metabolic, and cardiovascular parameters. Although the exclusion of healthy controls receiving regular medication may have reduced between-group medication-related confounding, the potential effects of antipsychotic treatment in the patient group could not be completely eliminated. To minimise this potential effect, efforts were made to account for antipsychotic treatment burden in the analyses using chlorpromazine-equivalent doses, and the main findings remained significant after these adjustments. Nevertheless, because the immunological and metabolic effects of different antipsychotics may vary, it could not be definitively determined within the scope of this study whether the observed alterations were entirely attributable to the biology of the disorder or to class-specific effects of the medications. In addition, the duration of antipsychotic treatment could not be included in the analyses. Furthermore, the cumulative number of psychotic episodes, which may reflect illness burden, was not included in the analyses.

The single-centre design and limited sample size restrict the generalizability of the findings, and the limited consideration of various clinical confounders in the analyses may have influenced the findings. In addition, cardiovascular risk was assessed using only the QRISK3 algorithm, and other advanced cardiovascular risk assessment methods could not be included in this study.

Only peripheral blood levels were assessed in this study, and other body fluids could not be included in the analyses. Cerebrospinal fluid and neural tissue analyses could not be performed, and the observed findings could not be replicated. Repeated measurements of peripheral blood cytokine levels throughout the day could not be performed, and cytokine alterations within neural tissue could not be evaluated. In addition, changes in monocyte subsets and TLR/HLA-DR expression were evaluated at the phenotypic level using flow cytometry; however, monocyte migration, endothelial repair capacity, markers of immunosenescence, and cellular functional responses were not directly assessed.

Potential comorbidities that may emerge during follow-up could not be addressed, and the severity of these psychiatric disorders could not be evaluated. Other confounding factors that may influence blood parameters (such as social determinants of health, alcohol and substance use, and additional socioeconomic variables) could not be included in the analyses. Obese and overweight participants were not examined in our study, and cardiovascular risk assessments specific to these individuals were not conducted. Psychometric tests (such as the Stroop Test and the Iowa Gambling Task) to assess participants’ cognitive functions were not administered, and neuroimaging could not be performed. Furthermore, the inclusion of only patients in remission with a total PANSS score below 70 resulted in a relatively clinically stable schizophrenia cohort, and patients with different levels of clinical severity were not represented in the study. Moreover, the assessment of only a limited set of immunological parameters may have restricted a comprehensive evaluation of the inflammatory process, and other biological mechanisms potentially related to cardiovascular risk in schizophrenia may have been overlooked.

## 5 Conclusion

In this study, compared with the control group, patients with schizophrenia exhibited higher IL-6 and IL-10 levels, lower NF-κB and IFN-γ levels, an increased proportion of classical monocytes, a decreased proportion of non-classical monocytes, and subtype-specific alterations in TLR-2/TLR-4 and HLA-DR expression. Additionally, compared with the control group, the schizophrenia group exhibited higher TG levels, QRISK3 scores, CIMT, and the prevalence of diastolic dysfunction, together with lower GLS and mitral E/A ratio, consistent with the pattern of subclinical cardiovascular alterations reported in schizophrenia. In addition, the QRISK3 score was positively correlated with mean CIMT and negatively correlated with IL-6 and TNF-α levels. However, regression analyses showed that IL-6 was positively associated with CIMT in participants with schizophrenia, whereas QRISK3 was not significantly associated with any of the inflammatory biomarkers. Hierarchical regression analysis also showed that increased IL-6 levels and decreased NF-κB levels were the biomarkers most strongly associated with a diagnosis of schizophrenia.

Our findings suggest that inflammatory cytokines may be altered in schizophrenia, that several traditional cardiovascular risk measures may be increased in individuals with schizophrenia, and that inflammatory biomarkers are not independently associated with the increased cardiovascular risk observed in schizophrenia. Further multicenter, longitudinal studies with larger sample sizes, including medication-free participants, are needed to better clarify this relationship and elucidate its underlying mechanisms.

## CRediT authorship contribution statement

**Muhammed Tuğrul Ergün:** Writing – review & editing, Writing – original draft, Visualization, Validation, Supervision, Methodology, Investigation, Formal analysis, Data curation, Conceptualization. **Memduha Aydın:** Writing – review & editing, Writing – original draft, Visualization, Validation, Supervision, Methodology, Investigation, Formal analysis, Data curation, Conceptualization. **Rukiye Tekdemir:** Writing – review & editing, Writing – original draft, Visualization, Validation, Supervision, Methodology, Investigation, Formal analysis, Data curation, Conceptualization. **Ömer Bayırlı:** Writing – review & editing, Writing – original draft, Investigation. **Kadri Murat Gürses:** Writing – review & editing, Writing – original draft, Investigation. **Muhammed Ulvi Yalçın:** Writing – review & editing, Writing – original draft, Investigation. **Halil Özalp:** Writing – review & editing, Writing – original draft, Investigation. **Hasibe Artaç:** Writing – review & editing, Writing – original draft, Investigation. **Ayça Ceylan:** Writing – review & editing, Writing – original draft, Investigation. **Sedat Abuşoğlu:** Writing – review & editing, Writing – original draft, Visualization, Validation, Methodology, Formal analysis, Data curation. **Fikret Akyürek:** Writing – review & editing, Writing – original draft, Visualization, Validation, Methodology, Formal analysis, Data curation. **Halil Güven:** Writing – review & editing, Writing – original draft, Investigation. **Yavuz Selvi:** Writing – review & editing, Writing – original draft, Visualization, Validation, Methodology, Formal analysis, Data curation. **Mustafa Esad Tezcan:** Writing – review & editing, Writing – original draft, Visualization, Validation, Supervision, Methodology, Investigation, Formal analysis, Data curation, Conceptualization.

## Ethics declaration

Written informed consent to take part in the study and to publish the article has been obtained from all participants or their legal representatives. The privacy rights of participants have been observed.

This study included organ or tissue donors. This study includes human biological material and consent was obtained by donors, or their next of kin or legal representatives, for use in this study and for publication of the article. The samples used in this research were not sourced from executed prisoners or prisoners of conscience.

This study was performed in compliance with relevant laws, regulatory frameworks and guidelines where the research took place. This study was approved by the Selçuk University Faculty of Medicine Ethics Committee. (Approval No. 2024/368)

## Funding

Funding for this study was provided by a grant from the Scientifc Research Project Coordination Unit of Selçuk University (Project no: 24122024).

## Declaration of competing interest

The authors have no conflict of interest to declare.

## Data Availability

Data will be made available on request.
